# Unified Brain Mechanisms Underlying the Processing of Word Order Permutations of Arguments and Adjuncts in Sentences

**DOI:** 10.1002/hbm.70604

**Published:** 2026-08-13

**Authors:** Daiki Asami, Taiga Naoe, Michiru Makuuchi, Masatoshi Koizumi

**Affiliations:** ^1^ National Institute of Informatics Chiyoda Tokyo Japan; ^2^ Medical Institute of Developmental Disabilities Research, Showa Medical University Tokyo Japan; ^3^ Section of Neuropsychology Research Institute of National Rehabilitation Center for Persons With Disabilities Tokorozawa Japan; ^4^ Department of Linguistics, Graduate School of Arts and Letters Tohoku University Sendai Japan

**Keywords:** adjuncts, arguments, fMRI, Japanese, scrambling, word order

## Abstract

Human language often allows flexible word order in sentences with either an argument or an adjunct scrambled. This study examined brain mechanisms during the comprehension of such sentences in Japanese by conducting a functional magnetic resonance imaging (fMRI) experiment. The imaging results revealed that regardless of whether arguments or adjuncts undergo scrambling, the comprehension of noncanonical word order sentences yields a higher brain activation than their canonical counterparts. Specifically, the activation was found in the left inferior frontal gyrus and the posterior superior temporal sulcus. The current results lend neurological support for the canonical versus noncanonical word order distinction in sentences with adjuncts, suggesting a unified explanation for the brain mechanisms for the processing of word order permutation in human language.

## Key Points

- Unified processing of scrambling
  ○ fMRI results showed that noncanonical sentences elicit stronger activation than canonical sentences in the left inferior frontal gyrus (LIFG) and left posterior superior temporal sulcus (pSTS), regardless of whether an argument or adjunct is scrambled, suggesting common neural mechanisms for word order permutation.
- Subject‐scrambling‐specific effects in left inferior frontal sulcus (LIFS)
  ○ Dislocated subjects, but not adjuncts, increased activation in the LIFS, indicating that scrambling of subjects requires additional processing costs compared to scrambling of adjuncts.
- Support for syntax‐based accounts
  ○ The findings confirm that canonical sentences with adjuncts are easier to process than their noncanonical counterparts, providing neurolinguistic support for the three‐way classification of adjuncts (VP, TP, and CP). The neural effects of syntactic complexities associated with scrambling are not fully reducible to predictive processing captured by a transformer‐based large language model.

## Graphical abstract

Noncanonical word order sentences elicited stronger activation than their canonical counterparts in the left inferior frontal gyrus and posterior superior temporal sulcus, regardless of whether arguments or adjuncts were scrambled, suggesting unified neural mechanisms for word‐order permutation that involve additional hierarchical structure building and filler–gap dependency formation.

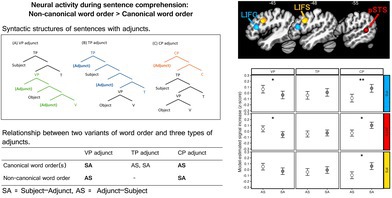

## 1 Introduction

Human language allows the displacement of phrases. This is represented by flexible word orders in many languages. In Japanese, for instance, transitive sentences can be ordered as either SOV (subject–object–verb) or OSV (object–subject–verb) without changing the basic meaning.

**Table:** 

(1)	a.	John‐ga	Mary‐o		osi‐ta.	[SOV (canonical)]
		John‐NOM	Mary‐ACC		push‐PST	
	b.	Mary‐o_1_	John‐ga	t_1_	osi‐ta.	[OSV (noncanonical)]
		Mary‐ACC	John‐NOM		push‐PST	
		“John pushed Mary.”				

Within the framework of generative linguistics (Chomsky 1965, 1981, 1995), it is widely assumed that the SOV is basic (i.e., canonical) while the OSV is nonbasic (i.e., noncanonical), derived by fronting the object over the subject as indicated by the t(race) (Hoji 1985; Saito 1985). The syntactic operation behind this fronting is referred to as scrambling (Ross 1967).^1^

**FIGURE 1:**
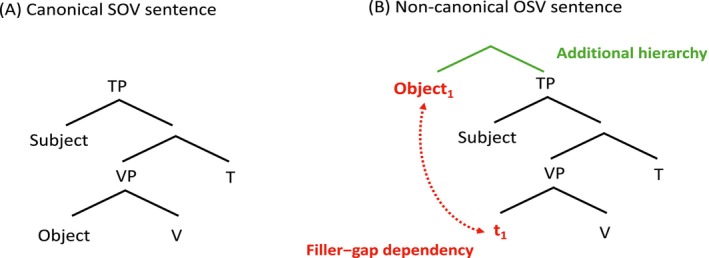
Tree structures of canonical SOV and noncanonical OSV sentences in Japanese. (A) The structure of canonical SOV sentences, whereas (B) The structure of noncanonical OSV sentences. The two structures are distinct in two aspects: (i) the scrambling of the object over the subject results in an additional hierarchical node in the OSV sentences; and (ii) the OSV sentences involve scrambling, yielding a filler–gap dependency between the scrambled object and its trace in its original position. These are believed to contribute to the processing difficulty in the OSV sentences compared to the SOV sentences.

Humans do not comprehend canonical sentences and noncanonical sentences with equal ease. Behavioral studies have provided converging evidence that canonical sentences enjoy a processing advantage over their noncanonical counterparts across different languages (e.g., for Finnish: Kaiser and Trueswell 2004; for Japanese: Tamaoka et al. 2005; for Kaqchikel: Koizumi et al. 2014; for Korean: Kim 2012; for Seediq: Ono et al. 2020; for Tongan: Tamaoka et al. 2024). For instance, Tamaoka et al. (2005) found that native speakers of Japanese took longer to process OSV order than SOV order, indicating that the former is harder to process than the latter. This finding follows from the general assumption in psycholinguistics that the more complex the linguistic representation is, the higher the cognitive cost is (e.g., Gibson 2000; Hawkins 2004; Koizumi 2023; Marantz 2005; O'Grady 1997; Pritchett and Whitman 1995). OSV sentences are representationally more complex than SOV sentences because the scrambling of the object creates an additional hierarchical node and filler–gap dependency (Figure 1).^2^ The latter yields a verbal working memory cost because of the maintenance of the scrambled phrase until its original position becomes clear. Thus, it is expected that OSV sentences are computationally more costly than their SOV counterparts. The same reasoning applies to the processing asymmetry between canonical and noncanonical word orders in other cases.

To uncover the neural processes behind the comprehension of flexible word order sentences, numerous studies have been conducted with a functional magnetic resonance imaging (fMRI) technique (e.g., for German: Bornkessel et al. 2005; Fiebach et al. 2005; Friederici et al. 2006; Makuuchi et al. 2013; for Japanese: Iwabuchi et al. 2019; Jeong et al. 2025; Kim et al. 2009; Kinno et al. 2008; Koizumi et al. 2012; for Kaqchikel: Koizumi and Kim 2016; Ohta et al. 2017). For example, Kim et al. (2009) revealed greater activity in the left inferior frontal gyrus (LIFG) when participants processed OSV sentences than when they processed SOV sentences in Japanese. This increased activity can be taken as evidence that the LIFG supports the mechanisms required to carry out processes for additional hierarchical structure building and filler‐gap dependency formation in OSV sentences (see, e.g., Friederici and Kotz 2003; Unger et al. 2023, among many others, for reviews of the role of the relevant region in sentence comprehension).

**FIGURE 2:**
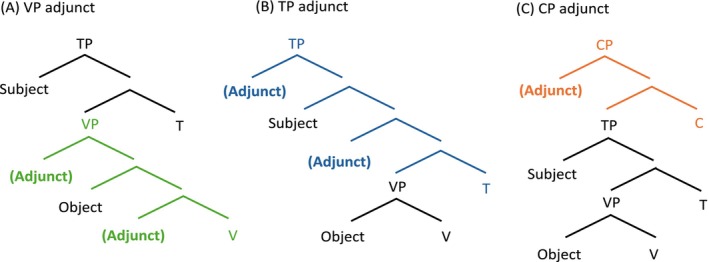
Original positions of three types of adjuncts. (A) The VP adjunct originates within the VP node; thus, it immediately precedes or follows the object. (B) The TP adjunct originates within the TP node; hence, it immediately precedes or follows the subject. (C) The CP adjunct originates within the CP node; thus, it appears at the sentence‐initial position. Note that the CP node may be projected in the tree structure even when no CP adjunct or overt C head is used in a sentence, although this is immaterial to the current study.

Importantly, sentences may include a nonargument phrase, namely, an adjunct (e.g., *tabun* “probably,” *kinoo* “yesterday,” and *yukkuri* “slowly”). Theoretical linguists hypothesize that a particular type of adjunct is affiliated with a specific projection in a clause structure (Asami 2024; Bruening 2013; Cinque 1999; Koizumi 1993; Pollard and Sag 1994). Under this hypothesis, we may postulate at least three types of adjuncts, namely, VP, TP, and CP adjuncts (cf. Koizumi 1993). Their hypothesized original positions are schematized in Figure 2. VP adjuncts (e.g., *yukkuri* “slowly”) originate within VP; TP adjuncts (e.g., *kinoo* “yesterday”) originate within TP, and CP adjuncts (e.g., *tabun* “probably”) originate within CP.

Under this three‐way classification of adjuncts, transitive sentences with each type of adjunct have canonical and noncanonical word orders. We illustrate this by taking AS(OV) and SA(OV) word orders as examples (“A” stands for “adjunct”). For VP adjuncts, SA is canonical, while AS is noncanonical and derived by scrambling the adjunct over the subject.^3^

**Table:** 

(2)	AS and SA sentences with VP adjuncts:					
a.	**Yukkuri**_**1**_	John‐ga	t_1_	Mary‐o	osi‐ta.	[AS (noncanonical)]
	**slowly**	John‐NOM		Mary‐ACC	push‐PST	
b.	John‐ga	**yukkuri**		Mary‐o	osi‐ta.	[SA (canonical)]
	John‐NOM	**slowly**		Mary‐ACC	push‐PST	
	“John pushed Mary slowly.”					

For TP adjuncts, both AS and SA are canonical.

**Table:** 

(3)	AS and SA sentences with TP adjuncts:				
a.	**Kinoo**	John‐ga	Mary‐o	osi‐ta.	[AS (canonical)]
	**yesterday**	John‐NOM	Mary‐ACC	push‐PST	
b.	John‐ga	**kinoo**	Mary‐o	osi‐ta.	[SA (canonical)]
	John‐NOM	**yesterday**	Mary‐ACC	push‐PST	
	“John pushed Mary yesterday.”				

For CP adjuncts, AS is canonical, while SA is noncanonical and derived by scrambling the subject over the adjunct.

**Table:** 

(4)	AS and SA sentences with CP adjuncts:					
a.	**Tabun**	John‐ga		Mary‐o	osi‐ta.	[AS (canonical)]
	**probably**	John‐NOM		Mary‐ACC	push‐PST	
b.	John‐ga_1_	**tabun**	t_1_	Mary‐o	osi‐ta.	[SA (noncanonical)]
	John‐NOM	**probably**		Mary‐ACC	push‐PST	
	“Probably, John pushed Mary.”					

Table 1 summarizes the relationship between the two word orders and three adjunct types.

**TABLE 1:** The relationship between the SA and AS word orders and the three types of adjuncts.

Adjunct type	Canonical word order(s)	Noncanonical word order
VP adjunct	SA	AS
TP adjunct	AS and SA	
CP adjunct	AS	SA

Conducting a processing time experiment, Koizumi and Tamaoka (2006) provided behavioral data consistent with this relationship. They found that it took longer to process noncanonical sentences with adjuncts than their canonical counterparts. In particular, A_VP_S sentences took longer to process than SA_VP_ sentences; there was no significant difference between A_TP_S and SA_TP_ sentences; and SA_CP_ sentences took longer to process than A_CP_S sentences (the subscripts indicate the adjunct type). This is exactly what the above classification would predict under the assumption that noncanonical word order sentences are representationally more complex (due to an additional hierarchical node and filler–gap dependency) and, consequently, computationally more costly than their canonical counterparts. Therefore, the findings by Koizumi and Tamaoka (2006) suggest that scrambling leads to a processing cost regardless of whether it targets an argument (i.e., SA_CP_ vs. A_CP_S) or an adjunct (i.e., A_VP_S vs. SA_VP_).

However, the processing time data in Koizumi and Tamaoka (2006) cannot tell us much about the cognitive processes behind the comprehension of the scrambling of adjuncts. In particular, their results raise the following research question: Does the scrambling of an adjunct and the scrambling of an argument involve similar or distinct neural mechanisms during comprehension? The present study aimed to shed light on this question by conducting an fMRI experiment in Japanese. Our experiment crossed two types of word orders (AS and SA) with three types of adjuncts (CP, TP, and VP adjuncts), allowing us to compare the argument scrambling (SA_CP_ vs. A_CP_S) and the adjunct scrambling (A_VP_S vs. SA_VP_). For the TP adjunct sentences, both SA_TP_ and A_TP_S are taken as canonical; hence, no difference is expected.

It is important to point out that during language comprehension, humans anticipate upcoming information, and when unexpected input occurs, processing difficulty arises; this effect can be operationalized with surprisal (i.e., negative log probability) estimates derived from language models (Hale 2001; Levy 2008). The strongest view on this matter holds that surprisal explains most, if not all, of processing difficulty (Levy 2008; Smith and Levy 2013). This view rests on the premise that a language model whose surprisal estimates more accurately predict neural or behavioral measures of comprehension is more closely aligned with human language mechanisms (Oseki 2023). A recent study reported that surprisal values derived from the transformer‐based language models (i.e., GPT‐2, Radford et al. 2019) better explained fMRI responses during sentence processing than grammar‐based models (Oh et al. 2022). Against this background, recent reports that transformer‐based language models such as GPT‐2 explain a portion of the variance in neural responses during sentence comprehension (e.g., Goldstein et al. 2022; Tuckute et al. 2024), together with the proposal that these models capture aspects of human predictive processing, warrant consideration. Here, we investigated whether neural activity associated with scrambling would remain even when taking into account the prediction that makes no explicit assumptions about syntax, as operationalized by GPT‐2 surprisal. This approach allowed us to examine whether scrambling effects reflect the processing costs of syntactic complexities rather than prediction‐related computations alone.

## 2 Methods

### 2.1 Participants

A total of 35 native Japanese speakers (24 women; 19–35 years old, mean = 23.80 years old, SD = 4.35) participated in the fMRI experiment for monetary compensation. They had no history of neuropsychiatric disorders, had normal or corrected‐to‐normal vision, and were right‐handed, as assessed by the Japanese‐translated version of the FLANDERS handedness questionnaire (Okubo et al. 2014) (range 70–100, mean = 98.00, SD = 6.23). The study was approved by the ethics committee of the National Rehabilitation Center for Persons with Disabilities in Japan. Written informed consent was obtained from each participant prior to the experiment.

### 2.2 Materials and Design

The experiment had a within‐subjects 2 × 3 factorial design. It manipulated WORD ORDER (AS vs. SA) and ADJUNCT (VP, TP, and CP adjuncts), yielding a total of six conditions: A_VP_S, SA_VP_, A_TP_S, SA_TP_, A_CP_S, and SA_CP_. Example sentences for each condition are provided below:

**Table:** 

(5)	A_VP_S and SA_VP_ sentences:				
a.	**Subayaku**	Satoo‐san‐ga	manga‐o	kai‐ta.	[A_VP_S]
	**quickly**	Sato‐Mr./Ms.‐NOM	comic‐ACC	draw‐PST	
b.	Satoo‐san‐ga	**subayaku**	manga‐o	kai‐ta.	[SA_VP_]
	Sato‐Mr./Ms.‐NOM	**quickly**	comic‐ACC	draw‐PST	
	“Mr./Ms. Sato drew a comic quickly.”				

(6)	A_TP_S and SA_TP_ sentences:				
a.	**Ototoi**	Satoo‐san‐ga	manga‐o	kai‐ta.	[A_TP_S]
	**two.days.ago**	Sato‐Mr./Ms.‐NOM	comic‐ACC	draw‐PST	
b.	Satoo‐san‐ga	**ototoi**	manga‐o	kai‐ta.	[SA_TP_]
	Sato‐Mr./Ms.‐NOM	**two.days.ago**	comic‐ACC	draw‐PST	
	“Mr./Ms. Sato drew a comic two days ago.”				

(7)	A_CP_S and SA_CP_ sentences:				
a.	**Tinamini**	Satoo‐san‐ga	manga‐o	kai‐ta.	[A_CP_S]
	**by.the.way**	Sato‐Mr./Ms.‐NOM	comic‐ACC	draw‐PST	
b.	Satoo‐san‐ga	**tinamini**	manga‐o	kai‐ta.	[SA_CP_]
	Sato‐Mr./Ms.‐NOM	**by.the.way**	comic‐ACC	draw‐PST	
	“By the way, Mr./Ms. Sato drew a comic.”				

Adjuncts were classified into three types based on a negation test (Koizumi 1993). According to Koizumi (1993), short negation in Japanese (*nai* “not”) can negate phrases contained within VP, while long negation (*wakedewanai* “it is not the case”) can negate phrases contained within TP. With respect to this distinction, VP, TP, and CP adjuncts behave differently as follows. VP adjuncts can be negated by both short and long negation, consistent with the view that they are affiliated with the VP domain.

**Table:** 

(8)	a.	[_VP_ **Subayaku**	hasir]‐**ana**‐katta.	
		**quickly**	run‐**NEG**‐PST	
		'(I) ran not quickly.'		
	b.	[_TP_ [_**VP**_ **Subayaku**	hasit]‐ta]	**wakedewanai**.
		**quickly**	run‐PST	**it.is.not.the.case**
	“The way in which (I) ran was not quick.”			

TP adjuncts, in contrast, can be negated by long negation but not by short negation, reflecting their affiliation with the TP layer.

**Table:** 

(9)	a.	[_TP_	[_VP_ hasir]‐**ana**‐katta].		
		**two.days.ago**	run‐**NEG**‐PST		
		Possible reading: “(I) did [not run] two days ago.”			
		Impossible reading: “(I) ran [not two days ago].”			
	b.	[_TP_ **Ototoi**	[_VP_ hasit]‐ta]	**wakedewanai.**	
		**two.days.ago**	run‐PST	**it.is.not.the.case**	
	“The time when (I) ran was [not two days ago].”				

Finally, CP adjuncts can be negated by neither short nor long negation, suggesting that they are merged with the CP level, which is outside of the TP domain.

**Table:** 

(10)	a.	[_CP_ **Tinamini**	[_TP_	[_VP_ hasir]‐**ana**‐katta]].		
		**by.the.way**		run‐**NEG**‐PST		
		Possible reading: “By the way, (I) did [not run].”				
		Impossible reading: “[Not by the way], (I) ran.”				
	b.	[_**CP**_ **Tinamini**	[_TP_	[_VP_ hasit]‐ta]	**wakedewanai**]	
		**by.the.way**		run‐PST	**it.is.not.the.case**	
		Possible reading: “By the way, it is not the case that I ran.”				
		Impossible reading: “[Not by the way], (I) ran.”				

The results of the negation test are summarized in Table 2.

**TABLE 2:** Results of a negation test for three types of adjuncts (VP, TP, and CP adjuncts).

Adjunct type	Can be targeted by short negation?	Can be targeted by long negation?
VP adjunct	Yes	Yes
TP adjunct	No	Yes
CP adjunct	No	No

Based on the negation test, we selected 20 items for each type of adjunct (VP, TP, and CP adjuncts) to construct the experimental materials. We created 40 sets of simple transitive sentences, each consisting of a subject, object, and verb. Subjects were 40 Japanese family names combined with the suffix *‐san* “Mr./Ms.”; objects were 40 common nouns; and verbs were 40 different verbs. Each base sentence was combined with an adjunct to create AS sentences. Each adjunct appeared twice across the stimuli. We then created corresponding SA sentences by swapping the adjunct and subject. This procedure generated a total of 240 sentences (40 sentences in six conditions each).

To account for the probability of each sentence, we derived negative log‐probability (i.e., surprisal) for each stimulus sentence from a GPT‐2 Medium model retrained on Japanese (https://huggingface.co/rinna/japanese‐gpt2‐medium, accessed on November 7, 2024, Sawada et al. 2024). In a previous study comparing how well surprisal values derived from different language models predict fMRI responses during sentence processing, GPT‐2 Medium was shown to outperform grammar‐based models as well as its larger versions (Oh et al. 2022). We defined sentence surprisal as the sum of the surprisal values for all tokens contained within each sentence. In the VP and TP adjunct stimuli, surprisal was higher for the SA word order than for the AS word order (*ps* < .05, Table 3), suggesting that the predictions of our hypothesis and the surprisal‐based hypothesis would not align for these conditions. In the CP adjunct stimuli, by contrast, surprisal was higher for the SA word order than for the AS word order (*p* < .001), suggesting that our hypothesis and the surprisal‐based account would make the same predictions in this condition.

**TABLE 3:** Summary statistics for sentence‐level negative log‐probabilities (surprisal, centered at the mean).

Adjunct type	AS	SA	t	p
VP adjunct	−0.570 (3.996)	−0.021 (4.038)	−2.234	0.031
TP adjunct	−0.707 (4.297)	1.287 (4.815)	−4.042	< 0.001
CP adjunct	−2.168 (4.756)	2.179 (4.421)	−5.742	< 0.001

*Note:* Sentence‐level surprisal was calculated as the sum of the surprisal values of all tokens within each sentence. Values are reported as means (SDs) for each combination of WORD ORDER and ADJUNCT. The reported *t* values were obtained from follow‐up pairwise comparisons of WORD ORDER within each level of ADJUNCT after a significant WORD ORDER × ADJUNCT interaction was identified in a two‐way repeated‐measures ANOVA with WORD ORDER and ADJUNCT as within‐item factors, using Type III sums of squares, *F*(2, 78) = 15.31, *p* < .001. Simple‐effects comparisons were performed using the emmeans function, and the resulting *p* values were adjusted across the three simple‐effect comparisons using the FDR procedure.

Note that the sum of token‐level surprisal values within each sentence can correlate with sentence length. In our stimuli, sentence surprisal was positively correlated with sentence token length (Pearson's *r* = 0.286, 95% CI = [0.166, 0.399], *p* < 0.0001). To ensure that the surprisal effect reflected contextual predictability rather than sentence length, we additionally conducted analyses using a length‐residualized surprisal measure, obtained as the residuals from a linear model with summed surprisal as the dependent variable and token size as the predictor (see Tables S1 and S2).

### 2.3 Procedure

After the fixation mark (*) for 700 ms and the blank for 100 ms, each sentence was presented with the rapid serial visual presentation paradigm (700 ms duration per word and 100 ms blank screen between the words). In 20% of trials, a probe sentence (e.g., *Arai‐san‐ga tabeta?* “Did Mr. Arai eat?”) was presented for 2 s after 100 ms from the offset of the last word of the sentence (i.e., a verb). During the probe presentation, participants judged if the content of a probe was consistent with the previously presented sentence by pressing the response buttons with their index finger (yes) or middle finger (no) as quickly as possible. The response hand (right vs. left) was counterbalanced across participants. Assignments of the probes were randomized for each session and for each participant. Half of the probes described the content of the preceding sentence, while the other half did not. The time from the offset of the target sentence to the onset of the next trial randomly varied between 7 and 9 s (average 8 s). Stimulus presentation was controlled by presentation software (Neurobehavioral Systems Inc., Albany, CA, USA).

### 2.4 fMRI Data Acquisition

The MRI data were collected with a 3 T MRI scanner (MAGNETOM Skyra; Siemens, Erlangen, Germany). We acquired T1‐weighted high‐resolution structural images (MPRAGE sequence, TR = 2300 ms, TE = 2.98 ms, inversion time = 900 ms, flip angle = 9°, field of view = 256 × 256 mm, matrix 256 × 256, sagittal 224 slices, 1 mm isotropic resolution). We obtained 2885 functional scans with a multiband echo‐planar imaging (EPI) sequence (repetition time = 1000 ms, echo time = 30 ms, flip angle = 68°, field of view = 192 × 192 mm, matrix 64 × 64, 30 axial slices, slice thickness = 3 mm with 1 mm gap, and multiband acceleration factor = 2) (Moeller et al. 2010). The slices were aligned to the AC–PC plane, covering the entire brain.

### 2.5 Preprocessing of fMRI Data

The functional images were preprocessed in SPM12 (http://www.fil.ion.ucl.ac.uk/spm/). All images were realigned to the mean image, and the difference in slice acquisition timing was corrected. Then, the functional images were spatially normalized in SPM12 using the EPI Montreal Neurological Institute (MNI) template in two steps: (i) estimating the normalization parameters and (ii) writing the normalized images with the obtained parameters. All functional images were transformed into the MNI stereotaxic space to allow multi‐subject analyses. The functional images were resampled into 3 × 3 × 3 mm^3^ voxels and smoothed with a 6 mm full‐width at half maximum (FWHM) Gaussian kernel.

### 2.6 fMRI Data Analysis

We processed the fMRI data using the SPM12 software package (http://www.fil.ion.ucl.ac.uk/spm/). We estimated the condition effects in each voxel per participant with a general linear model. The design matrix included the 13 regressors for the sentences of the six conditions, the probes with correct answers of the six conditions, and a condition for the probes with wrong answers. Sentence‐level GPT2‐surprisal and the six rigid‐body motion parameters were included as covariates of no interest. We removed low‐frequency noise using a high‐pass filter with a cut‐off period of 128 s. We estimated temporal correlations in the fMRI time series using an autoregressive AR(1) model in the estimation of parameters to correct for non‐sphericity during statistical inference. We conducted a whole‐brain random‐effects analysis at the group level. Main effects and planned interaction contrasts (AS vs. SA within each ADJUNCT condition) were tested using *t*‐tests. Statistical maps were first thresholded at the voxel level at *p* < 0.001 (uncorrected), and clusters exceeding 100 contiguous voxels were retained. Cluster‐level significance was assessed using false discovery rate (FDR) correction, with a threshold of *q* < 0.05.

We conducted the region of interest (ROI) analyses on the LIFG, the left inferior frontal sulcus (LIFS), and the posterior superior temporal sulcus (pSTS). We focused on these three regions because they were shown to be activated by scrambling of arguments in previous fMRI studies (Iwabuchi et al. 2019; Kim et al. 2009; Kinno et al. 2008; Koizumi et al. 2012; Makuuchi et al. 2013). We defined the coordinates for the ROI creation as follows: LIFG (−46, 28, 13), LIFS (−52, 8, 28), and pSTS (−54, −51, 6). These were the centers of mass in Iwabuchi et al. (2019), which examined the effect of scrambling of arguments in Japanese.^4^ Using these coordinates as reference points, we generated subject‐specific ROIs in the following steps to account for inter‐individual variability in whether a spatially homologous location was functionally engaged as part of the language processing network. We created a 10 mm radius sphere centered on the individual local maximum that was both nearest to and within a radius of 10 mm centered on the predefined coordinates above. We determined the individual local maxima based on the contrast of experimental sentences versus rest (uncorrected *p* < 0.05). The centroids of the ROIs created in this manner were as follows: LIFG (−45.36, 26.99, 14.04), LIFS (−48.25, 8.41, 31.36), and pSTS (−54.82, −47.67, 6.46). On the Montreal Neurological Institute's 152 brain template (MNI152), the LIFG is located in the posterior part of the pars triangularis and includes portions of the LIFS and the anterior part of the pars opercularis. The ROI of the LIFS ranges from the LIFS through the junction with the inferior precentral sulcus to the most anterior part of the precentral gyrus. The pSTS was centered within the superior temporal sulcus and contained the middle temporal gyrus and superior temporal gyrus on the MNI152.

We extracted time series data for each participant's preprocessed data (realigned, slice timing corrected, co‐registered, and normalized) from these ROIs using Marsbar 0.44 (https://marsbar‐toolbox.github.io/). Using SPM, we applied high‐pass filtering (cut‐off period: 128 s) and linear detrends. We then estimated trial time courses (TTCs) using a general linear model with a finite‐impulse‐response (FIR) basis following previous studies (Iwabuchi et al. 2019; Makuuchi et al. 2013). However, to obtain trial‐by‐trial TTCs, we employed a least‐squares separate (LSS) design, in contrast to previous work that estimated condition‐averaged TTCs. Concretely, for each trial, we fit a design matrix with FIR regressors for the target trial and for all remaining trials, together with six motion covariates. The FIR basis spanned 0–15 s in 1‐s steps. Parameters were estimated via the Moore–Penrose pseudoinverse, and the first 16 coefficients were taken as the single‐trial TTC. We compared the magnitudes of the estimated TTC peaks. First, we calculated the TTC peak latency for each ROI and defined the time windows for statistical analysis (LIFG: 5–6 s, LIFS: 6 s, pSTS: 6 s after the onset of the sentences) based on their 95% confidence intervals (LIFG: 4.90–6.24, LIFS: 5.16–6.24, and pSTS: 5.34–6.10; Iwabuchi et al. 2019). The mean TTC plots are provided in Figure S3.

In the statistical analyses, we excluded trials containing the probe task (20% of all trials) to minimize potential confounding between neural activity related to syntactic building and task‐demand‐related responses. To test our hypothesis, we then conducted a mixed‐effects model analysis for each ROI. Specifically, we included the main effects and interactions of WORD ORDER (AS/SA), ADJUNCT (VP/TP/CP), and GPT2‐surprisal as fixed effects. Using likelihood‐ratio tests, we confirmed that adding GPT2‐surprisal and its interactions with WORD ORDER and ADJUNCT (Signal Increase ~ WORD ORDER × ADJUNCT × GPT2‐surprisal) significantly improved model fit relative to models without surprisal interactions (Signal Increase ~ WORD ORDER × ADJUNCT + GPT2‐surprisal) and without the interaction of WORD ORDER and ADJUNCT (Signal Increase ~ GPT2‐surprisal). Model‐comparison results for these fixed effects and interaction terms are provided in Supporting Information 5.^5^ For the random‐effects structure, we initially specified a maximal random‐effects structure justified by the design, including by‐participant random intercepts and random slopes for the within‐participant effects, as well as by‐item random intercepts (Barr et al. 2013). The random‐effects structure was simplified when models failed to converge or exhibited singular (boundary) fits, indicating that one or more variance components were estimated at (near) zero (Jaeger 2009). In the final models, we included by‐participant and by‐item random intercepts for LIFG and pSTS, whereas for LIFS, we included only a by‐participant random intercept. To verify that the models met commonly used diagnostic criteria for fixed‐effect multicollinearity, convergence, boundary (singular) fits, and clustering, we performed the following checks. We assessed fixed‐effect multicollinearity by computing variance inflation factors (VIFs) for all fixed‐effect terms, using a conventional threshold of VIF < 5 as indicating negligible collinearity. Convergence was evaluated using gradient‐based diagnostics, requiring the maximum absolute gradient to be sufficiently small. We further assessed boundary (singular) fits with the Singular function to confirm that variance components were not estimated at (near) zero. All reported models satisfied these diagnostic criteria (low VIFs, successful convergence, no evidence of singular fit). We also computed intraclass correlation coefficients (ICCs) to quantify the degree of clustering attributable to the random‐effects structure. The full diagnostic outputs are provided in Supporting Information 5.

We set the contrasts for unordered factors to sum to zero. Specifically, the contrasts for WORD ORDER were defined such that AS was coded as 1 and SA as −1. For ADJUNCT, we compared VP and CP against TP: the first contrast compared CP versus TP (with CP coded as 1, VP as 0, and TP as −1), while the second contrast compared VP versus TP (with CP coded as 0, VP as 1, and TP as −1). We treated TP as the reference (control) level because TP adjuncts were canonical in both the AS and SA conditions, whereas CP and VP adjuncts included noncanonical word‐order realizations; thus, TP provided a baseline against which the effects of CP and VP could be interpreted as deviations associated with noncanonical order. We conducted the statistical analysis using R version 4.4.2 (R Core Team 2024), employing the *lme4* package (Bates et al. 2015) for model fitting, the *performance* package (Lüdecke et al. 2021) for model diagnostics, the *lmerTest* package (Kuznetsova et al. 2017) for statistical tests, and the *emmeans* package (Lenth 2024) for post hoc comparisons. For post hoc tests following significant interaction effects, reported *p* values were corrected for multiple comparisons using the FDR procedure.

## 3 Results

The purpose of the behavioral task in this study was to maintain participants' engagement with the sentence stimuli. Implementing the task on every trial would have increased the duration of each trial and, consequently, the overall scanning session. We therefore presented the task on a random 20% subset of trials. Consequently, each participant contributed only 48 behavioral trials in total (8 trials per condition), which we considered insufficient for reliable inferential statistical analyses. Summary descriptive statistics for accuracy and reaction time are provided in Tables S3–S5.

**FIGURE 3:**
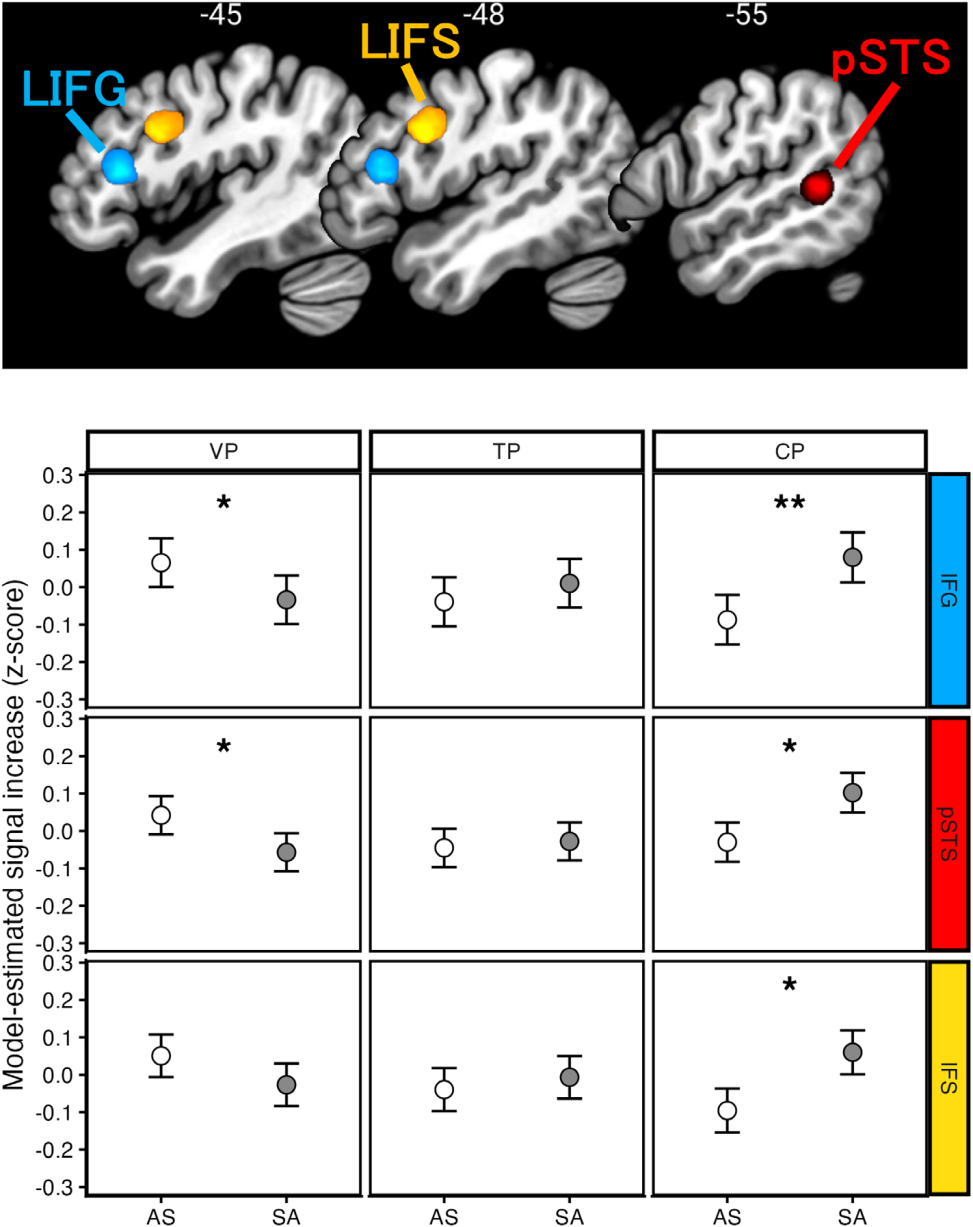
Top: ROI images with 50% population (i.e., voxels that were included in more than 15 of 30 participants' individual ROIs) were overlaid onto sagittal slices with *X* coordinates (millimeters) in MNI standard space as follows: Sky blue, left inferior frontal gyrus (LIFG); yellow, the left inferior frontal sulcus (LIFS); red, posterior superior temporal sulcus (pSTS). Bottom: The dot plots show the z‐scored peak values of the trial time courses (TTCs) in each ROI, estimated using linear mixed‐effects models. Error bars indicate the standard errors of the model estimates. “*” and “**” indicate that the signal significantly changes according to WORD ORDER within each ADJUNCT at *p* < 0.05 and *p* < 0.01 (FDR‐corrected *p* values), respectively. AS, adjunct–subject–object–verb word order; SA, subject–adjunct–object–verb word order.

We excluded five participants from the fMRI data analysis owing to low accuracy in answering probe questions (below 60%). Consequently, the analysis included 30 participants (21 women; 19–35 years old, mean = 24.43 years old, SD = 4.68). In the whole‐brain analysis, we found no significant WORD ORDER × ADJUNCT interaction contrasts. Results for the main effect of ADJUNCT, as well as condition‐specific activations relative to baseline, are reported in Figures S1 and S2 and Table S6. The ROI images relevant to the current study, along with the estimated mean signal increase in the ROIs, are presented in Figure 3. The full results of the mixed‐effects model analysis can be found in Supporting Information 5.

Across all three ROIs (LIFG, LIFS, and pSTS), we observed a significant WORD ORDER and ADJUNCT interaction consistent with our a priori hypothesis (LIFG: WORD ORDER: ADJUNCT [CP–TP], β = −0.062, 95% CI = [−0.098, −0.025], *t* = −3.317, *p* < 0.001; WORD ORDER: ADJUNCT [VP–TP], β = 0.068, 95% CI = [0.032, 0.101], *t* = 3.795, *p* < 0.001; LIFS: WORD ORDER: ADJUNCT [CP–TP], β = −0.064, 95% CI = [−0.105, −0.023], *t* = −3.054, *p* = 0.002; WORD ORDER: ADJUNCT [VP–TP], β = 0.062, 95% CI = [0.023, 0.101], *t* = 3.088, *p* = 0.002; pSTS: WORD ORDER: ADJUNCT [CP–TP], β = −0.054, 95% CI = [−0.090, −0.018], *t* = −2.923, *p* = 0.004; WORD ORDER: ADJUNCT [VP–TP], β = 0.051, 95% CI = [0.020, 0.089], *t* = 3.086, *p* = 0.002). In the LIFG and pSTS, we found the higher signal increases in the A_VP_S condition than in the SA_VP_ condition (LIFG: Cohen's *d* = 0.104, 95% CI = [0.014, 0.193], *z* = 2.265, *p* < 0.05; pSTS: Cohen's *d* = 0.100, 95% CI = [0.011, 0.190], *z* = 2.195, *p* < 0.05). In the same ROIs, we also found the higher signal increases in the SA_CP_ condition than in the A_CP_S condition (LIFG: Cohen's *d* = −0.174, 95% CI = [−0.274, −0.075], *z* = −3.443, *p* < 0.01; pSTS: Cohen's *d* = −0.134, 95% CI = [−0.233, −0.035], *z* = −2.662, *p* < 0.05). In the LIFS, we found the higher signal increase in the SA_CP_ condition than in the A_CP_S condition (Cohen's *d* = −0.160, 95% CI = [−0.258, −0.061], *z* = −3.189, *p* < 0.01).

Across all three ROIs (LIFG, LIFS, and pSTS), the interaction between ADJUNCT and GPT2‐surprisal was significant (LIFG: GPT2‐surprisal: ADJUNCT [CP–TP], β = −0.054, 95% CI = [−0.089, −0.018], *t* = −2.977, *p* = 0.003, [VP–TP], β = 0.056, 95% CI = [0.018, 0.094], *t* = 2.882, *p* = 0.004; LIFS: GPT2‐surprisal: ADJUNCT [CP–TP], β = −0.081, 95% CI = [−0.121, −0.041], *t* = −3.997, *p* < 0.001, [VP–TP], β = 0.046, 95% CI = [0.003, 0.088], *t* = 2.114, *p* = 0.035; pSTS: GPT2‐surprisal: ADJUNCT [CP–TP], β = −0.051, 95% CI = [−0.086, −0.016], *t* = −2.858, *p* = 0.004). We fitted a linear mixed‐effects model to examine the slope of GPT2‐surprisal for each level of ADJUNCT. The significant GPT2‐surprisal slope was observed in LIFG with VP adjunct and LIFS with CP adjunct (Tables 4, 5). In the LIFG, there was a significantly positive slope for the sentences with VP adjuncts (standardized β = 0.070, 95% CI = [0.019, 0.121], *z* = 2.687, *p* < 0.05). In the LIFS, there was a significantly negative slope for the sentences with CP adjuncts (standardized β = −0.084, 95% CI = [−0.132, −0.037], *z* = −3.465, *p* < 0.01). The interaction between ADJUNCT and GPT2‐surprisal and the surprisal slope within each ADJUNCT in LIFG and LIFS were consistently observed even when the effect of token size was regressed out from the GPT2‐surprisal (Supporting Information 5). Thus, these findings indicate that the surprisal effect reflected contextual predictability rather than sentence length.

**TABLE 4:** Estimated slope of surprisal‐related fMRI signal increase in the left inferior frontal gyrus (LIFG) for each adjunct condition.

	Standardized *β*	95% CI‐LL	95% CI‐UL	*z*	*p*
VP adjunct	**0.0697**	**0.0189**	**0.1206**	**2.6870**	**0.0216**
TP adjunct	0.0117	−0.0333	0.0567	0.5086	0.6110
CP adjunct	−0.0397	−0.0839	0.0046	−1.7583	0.1181

*Note:* *β* denotes the standardized slope. Bold values indicate statistically significant slopes based on FDR‐corrected *p* values.

Abbreviations: CI, confidence interval; LL, lower limit; UL, upper limit.

**TABLE 5:** Estimated slope of surprisal‐related fMRI signal increase in the left inferior frontal sulcus (LIFS) for each adjunct condition.

	Standardized *β*	95% CI‐LL	95% CI‐UL	*z*	*p*
VP adjunct	0.0426	−0.0111	0.0964	1.5539	0.1803
TP adjunct	0.0324	−0.0154	0.0802	1.3294	0.1837
CP adjunct	**−0.0840**	**−0.1315**	**−0.0365**	**−3.4649**	**0.0015**

*Note:* *β* denotes the standardized slope. Bold values indicate statistically significant slopes based on FDR‐corrected *p* values.

Abbreviations: CI, confidence interval; LL, lower limit; UL, upper limit.

## 4 Discussion

We conducted an fMRI experiment to elucidate the neural processes involved in the comprehension of canonical and noncanonical sentences with adjuncts. Our design crossed two types of word order (AS and SA) with three types of adjuncts (VP, TP, and CP adjuncts). The imaging results revealed that the comprehension of canonical versus noncanonical sentences elicited higher activation in the LIFG and the pSTS regardless of whether an argument or an adjunct was scrambled (i.e., SA_CP_ vs. A_CP_S; A_VP_S vs. SA_VP_). In contrast, enhanced activation in the LIFS was observed only when an argument was scrambled (i.e., SA_CP_ vs. A_CP_S). No significant activation was found in any regions of interest in the comparison between A_TP_S and SA_TP_. Finally, we confirmed that the word order effects observed for each adjunct were not fully accounted for by surprisal derived from a transformer‐based large language model (GPT‐2). We discuss the implications of these findings below.

The observed activation in the LIFG is consistent with findings from the previous fMRI studies focusing on the processing of noncanonical sentences with arguments scrambled in Japanese (Iwabuchi et al. 2019; Jeong et al. 2025; Kim et al. 2009; Kinno et al. 2008; Koizumi et al. 2012). These studies reported greater activation in the region corresponding to the LIFG for noncanonical word order sentences compared to their canonical counterparts. A body of evidence suggests that this region constitutes a core area for syntactic processing: syntactic complexity due to multiple syntactic embedding increased its activation (Makuuchi et al. 2009, 2013; den Ouden et al. 2012; Pallier et al. 2011; Wang et al. 2021); artificial grammar experiments have demonstrated enhanced activation of this region as a function of grammatical complexity (Chen et al. 2019, 2021; Petersson et al. 2012); and three‐word phrases elicited stronger activation than nonstructured three‐word lists (Zaccarella et al. 2017; Zaccarella and Friederici 2015). In our findings, the activation of the LIFG was observed regardless of whether an argument or an adjunct was scrambled. We take this to suggest that noncanonical sentences, irrespective of the type of scrambled constituent, always involve additional syntactic complexity.

One could raise the possibility that the increased activation in the LIFG for noncanonical sentences reflects differences in usage frequency rather than syntactic complexity. Specifically, less frequent configurations could lead to higher activation in the relevant region under the assumption that noncanonical word orders are less frequent than their canonical counterparts. However, the relationship between frequency and neural activation is not straightforward. Cross‐linguistic evidence shows that more frequent word orders do not necessarily result in reduced activation. For example, in Kaqchikel (a Mayan language spoken in Guatemala), the syntactically derived, noncanonical SVO order is more frequent in usage than the syntactically basic, canonical VOS order; yet, stronger LIFG activation was reported for SVO than for VOS (Koizumi and Kim 2016). In addition, the present study considered surprisal estimates, which are closely related to frequency, and found that the critical word order effects remained after controlling for this factor. Taken together, it is reasonable to conclude that frequency‐related factors are unlikely to fully account for the activation pattern in the LIFG.

Another possible interpretation of the observed activation in the LIFG is that it reflects semantic or pragmatic demands associated with information structure. We contend that this perspective is also unlikely to fully account for the current activation pattern, based on recent neuroimaging evidence provided by Jeong et al. (2025). The authors manipulated word order (SOV vs. OSV) and information structure (given–new vs. new–given) and reported that the corresponding region is sensitive to information‐structural demands. However, their findings suggest that activity in this region does not completely disappear even when information‐structural demands are controlled for in the processing of noncanonical sentences. Therefore, the purely informational structural account of the LIFG activation is not fully satisfactory. Clearly, further research is required that manipulates information structure in different word order sentences with adjuncts.

Comprehension of the noncanonical sentences also elicited higher activation in the pSTS compared to their canonical counterparts, irrespective of whether an argument or an adjunct was scrambled. Iwabuchi et al. (2019) and Jeong et al. (2025) both reported that noncanonical OSV sentences in Japanese enhanced activation in the region corresponding to the pSTS compared to canonical SOV sentences, but they arrived at different conclusions. Iwabuchi et al. (2019) proposed that the pSTS is involved in hierarchical structure building. The current result is compatible with this perspective under the assumption that scrambling requires additional hierarchical structure building regardless of whether arguments or adjuncts are scrambled.

In contrast, Jeong et al. (2025) claimed that this region is responsible for the assignment of verb‐related thematic roles (e.g., patient) in noncanonical sentences. This account can explain the higher activation in the pSTS for SA_CP_ sentences than for A_CP_S sentences under the theoretical assumption that subjects are thematically related to verbs either directly (e.g., Fukui 1986; Kitagawa 1986; Koopman and Sportiche 1988; Kuroda 1988) or indirectly via another linguistic element (e.g., Asami and Bruening 2025; Chomsky 1995; Kratzer 1996; Marantz 1984). However, this account is seemingly incompatible with the observed activation pattern in the comparison of A_VP_S and SA_VP_ sentences, because scrambled VP adjuncts have no direct or indirect thematic‐role relation with verbs. We suggest that Jeong et al.'s account can be made compatible with the present result with the following refinement: the pSTS supports the licensing of scrambled verb‐related phrases in their original positions. The departure of this revised account from Jeong et al.'s is that the relevant licensing process is not limited to arguments that have a direct or indirect thematic‐role relation with the verb but also extends to adjuncts that do not. This refined account is consistent with the observed activation pattern: since VP adjuncts must be semantically and syntactically licensed in relation to the verb in their original position, their displacement increases activation in the pSTS. Therefore, the present finding is compatible with either of the two existing accounts by Iwabuchi et al. (2019) and Jeong et al. (2025). Regardless of which account turns out to be correct in future research, the current result can be taken as evidence that similar mechanisms underlie the processing of noncanonical sentences with arguments or adjuncts scrambled.

Let us now turn to the LIFS. In contrast to the LIFG and pSTS, we observed a mixed pattern of activation in this region: noncanonical sentences increased activation in this region when a subject was scrambled (i.e., SA_CP_ vs. A_CP_S) but not when an adjunct was scrambled (i.e., A_VP_S vs. SA_VP_). One possible explanation for this asymmetry is that scrambling of an argument imposes greater working‐memory demands associated with dependency formation than scrambling of an adjunct. When a subject NP is displaced from its canonical position, it must be maintained in working memory until it is retrieved and integrated into the verb's argument structure. This process is taxing for working memory. Consistent with this interpretation, previous fMRI studies demonstrated that activity in the LIFS is associated with the distance of dependencies, reflecting the working‐memory costs required to maintain and retrieve constituents (Amici et al. 2007; Makuuchi et al. 2009, 2013). In contrast, a VP adjunct, being an optional modifier, is not part of the verb's argument structure. Consequently, the parser can start retrieving it from working memory and interpreting it with the developing structure before the verb's argument structure becomes clear during comprehension. As a result, its displacement may require relatively less maintenance or retrieval costs than displaced arguments. Under this view, scrambling of a subject would impose greater dependency‐related working‐memory demands than scrambling of a VP adjunct, which in turn could explain why increased activation in the LIFS was observed only when an argument was scrambled in the present experiment. Admittedly, this hypothesis remains speculative at this point and needs to be confirmed in future research.

Another possible interpretation of the current mixed results concerns the distinction between transitive subjects and VP adjuncts with respect to their relation to the verb. As noted above, some theoretical linguistic studies argue that the subject of a transitive verb is indirectly related to the verb (e.g., Asami and Bruening 2025; Chomsky 1995; Kratzer 1996; Marantz 1984). In contrast, VP adjuncts are generally assumed to be more directly associated with the verb (e.g., Koizumi 1993). Given this difference, the observed contrast between transitive subjects and VP adjuncts may arise because the processing of displaced transitive subjects requires additional processing demands due to their relatively indirect relation with the verb, resulting in higher LIFS activation. If this interpretation is correct, the mixed activation pattern under discussion may reflect whether displaced phrases are directly or indirectly associated with the verb.

One way to further investigate this possibility would be to examine how scrambling of surface subjects in unaccusative or passive constructions affects the activation in the LIFG. Theoretical studies have suggested that, in Japanese, subjects of passive and unaccusative constructions may originate as direct objects (e.g., Asami 2025; Jo and Seo 2023; Ishizuka 2012 for passive subjects; Asami and Bruening 2025; Fukuda 2017; Miyagawa 1989 for unaccusative subjects). Recent behavioral studies by Asami and Tomioka (2025, 2026) reported that the optional movement of unaccusative subjects incurs processing costs closely paralleling those for the scrambling of transitive objects. Extending their behavioral experiments to the present fMRI paradigm would allow a direct test of how displacement of verb‐(un)related phrases contributes to the LIFG activation.

In contrast to AS and SA sentences with VP or CP adjuncts, the comparison between AS and SA sentences with TP adjuncts did not reveal significant activation in any ROIs. This result is consistent with the view that both AS and SA word orders are canonical for sentences with TP adjuncts (Koizumi 1993).

The effect of word order within each adjunct remained significant even when the effect of surprisal was considered. This result suggests that the influence of scrambling on neural activity is not fully accounted for by contextual probability. Recent studies have shown that surprisal values derived from transformer‐based language models such as GPT‐2 explain substantial variance in neural activity during comprehension (e.g., Goldstein et al. 2022; Tuckute et al. 2024) and outperform grammar‐based models (Oh et al. 2022). These findings suggest that probabilities estimated by language models such as GPT, which make no explicit assumptions about syntax, capture at least some aspects of human predictive mechanisms during language processing (cf. Lopopolo et al. 2017; Shain et al. 2020). Under the assumption that language models whose surprisal estimates better predict neural or behavioral indices of comprehension are more closely aligned with human language mechanisms (Oseki 2023), this line of work has motivated the view that explicit syntactic principles may not be required to explain human language processing and, in some accounts, even language acquisition (Piantadosi 2024). Our results, however, indicate that linguistically motivated assumptions are essential for explaining neural responses during sentence comprehension, and our several additional findings are also consistent with this view. Both the neural contrasts predicted from condition differences in surprisal values and the observed direction of surprisal effects on neural activity diverged from the predictions of our syntax‐based account. Moreover, models including word order and adjunct type provided a better fit to the neural data than models including GPT2‐surprisal alone.

Overall, the results reveal a processing advantage for canonical sentences with adjuncts over their noncanonical counterparts. This finding aligns with the results of the behavioral study by Koizumi and Tamaoka (2006). Importantly, our fMRI experiment advances this prior work by shedding new light on the neural processes underlying the comprehension of noncanonical sentences with adjuncts. We found that both the LIFG and pSTS were engaged during the comprehension of noncanonical sentences involving scrambled arguments and adjuncts, suggesting that these structures require additional syntactic complexities. In contrast, the LIFS exhibited a mixed pattern: scrambling of subjects increased activation in this region, whereas scrambling of VP adjuncts did not. We attributed this pattern to different dependency‐related working‐memory demands or different degrees of relations between displaced phrases and verbs. Importantly, the effects of word order within each adjunct type were significant even after controlling for surprisal, indicating that these effects cannot be fully reduced to contextual predictability alone.

Before concluding this paper, let us finally discuss the implications of the current results for theoretical linguistics. The observed processing asymmetry between canonical and noncanonical sentences with adjuncts follows from the theoretically defined three‐way classification of adjuncts, according to which there are at least three types of adjuncts, each associated with the VP, TP, and CP layers of clause structure (cf. Koizumi 1993). The present results are consistent with this classification. More broadly, they are compatible with the view that particular adjuncts are affiliated with specific syntactic projections (Asami 2024; Bruening 2013; Cinque 1999; Koizumi 1993; Pollard and Sag 1994) but not with accounts that downplay, if not reject, the role of syntactic structure in adjunct licensing (e.g., Ernst 2002).

## 5 Conclusion

This study investigated the neural mechanisms underlying the comprehension of canonical versus noncanonical sentences with adjuncts in Japanese. The imaging results revealed that activation in the LIFG and pSTS was enhanced regardless of whether an argument or an adjunct was scrambled. The findings suggest that additional hierarchical structure building and filler–gap dependency formation are involved independently of the type of constituent scrambled. Future research may explore the neural basis of the comprehension of canonical versus noncanonical sentences with different types of adjuncts.

## Funding

This work was supported by JSPS KAKENHI Grant Numbers JP19H00532, JP19H05589, JP24H00085, JP24K16067, JP26K00006, and JP26K00007, and Japan Science and Technology Agency Moonshot R&D JPMJMS2292.

## Ethics Statement

The study was approved by the Ethics Committee of the National Rehabilitation Center for Persons with Disabilities in Japan.

## Consent

Written informed consent was obtained from each participant prior to the experiment.

## Conflicts of Interest

The authors declare no conflicts of interest.

## Supporting information


**Table S1:** Token size of each sentence.
**Table S2:** Sentence‐level negative log‐probabilities (surprisal) residualized for token size.
**Table S3:** Within‐participant mean accuracy across all participants.
**Table S4:** Within‐participant mean accuracy of participants included in the fMRI analysis (≥ 60% accuracy).
**Table S5:** Within‐participant mean RT (*Z*‐score) of participants included in the fMRI analysis (≥ 60% accuracy).
**Table S6:** Brain regions identified by the contrast of CP and VP > TP.
**Figure S1:** Results of a whole‐brain random‐effects analysis testing effects of ADJUNCT. Brain regions exhibiting marginally significant greater activation for CP and VP adjuncts than for TP adjuncts (CP and VP > TP, Cluster‐level FWE‐corrected *ps* < 0.05, Cluster‐level FDR‐corrected *qs* < 0.10) are overlaid on a rendered cortical surface (A) and on axial (B‐left) and sagittal (B‐right) slices (*X* = −15, *Y* = 8, *Z* = 5).
**Figure S2:** Brain images show results of whole‐brain random effects analyses (Each condition > Rest, voxel‐wise FDR‐corrected *ps* < 0.05).
**Figure S3:** Mean trial time course (TTC) plots for each condition in each ROI. Time zero and the black rectangles at the bottom of the TTC plots correspond to the onset and duration of sentence presentation, respectively. The light gray bands indicate the 95% confidence intervals of mean peak latency within each ROI. AS, adjunct–subject–object–verb word order; LIFG, left inferior frontal gyrus; LIFS, left inferior frontal sulcus; pSTS, posterior superior temporal sulcus; SA, subject–adjunct–object–verb word order.
**Table S7:** Likelihood‐ratio tests for fixed‐effect structure: LIFG.
**Table S8:** Fixed‐effect multicollinearity diagnostics of the final model: LIFG.
**Table S9:** Convergence, singularity, and clustering diagnostic of the final model: LIFG.
**Table S10:** Mixed‐effects model results: LIFG.
**Table S11:** The effect of WO within each ADJ: LIFG.
**Table S12:** Likelihood‐ratio tests for fixed‐effect structure: pSTS.
**Table S13:** Fixed‐effect multicollinearity diagnostics: pSTS.
**Table S14:** Convergence, singularity, and clustering diagnostic: pSTS.
**Table S15:** Mixed‐effects model results: pSTS.
**Table S16:** The effect of WO within each ADJ: pSTS.
**Table S17:** Estimated slope of surprisal‐related fMRI signal increase in the pSTS.
**Table S18:** Likelihood‐ratio tests for fixed‐effect structure: LIFS.
**Table S19:** Fixed‐effect multicollinearity diagnostics: LIFS.
**Table S20:** Convergence, singularity, and clustering diagnostic: LIFS.
**Table S21:** Mixed‐effects model results: LIFS.
**Table S22:** The effect of WO within each ADJ: LIFS.
**Table S23:** Likelihood‐ratio tests for fixed‐effect structure: LIFG (Model Using GPT Surprisal With the Effect of Token Size Regressed Out).
**Table S24:** Fixed‐effect multicollinearity diagnostics: LIFG (Model Using GPT Surprisal With the Effect of Token Size Regressed Out).
**Table S25:** Convergence, singularity, and clustering diagnostic: LIFG (Model Using GPT Surprisal With the Effect of Token Size Regressed Out).
**Table S26:** Mixed‐effects model results: LIFG (Model Using GPT Surprisal With the Effect of Token Size Regressed Out).
**Table S27:** The effect of WO within each ADJ: LIFG (Model Using GPT Surprisal With the Effect of Token Size Regressed Out).
**Table S28:** Estimated slope of surprisal‐related fMRI signal increase in the LIFG (Model Using GPT Surprisal With the Effect of Token Size Regressed Out).
**Table S29:** Likelihood‐ratio tests for fixed‐effect structure: pSTS (Model Using GPT Surprisal With the Effect of Token Size Regressed Out).
**Table S30:** Fixed‐effect multicollinearity diagnostics: pSTS (Model Using GPT Surprisal With the Effect of Token Size Regressed Out).
**Table S31:** Convergence, singularity, and clustering diagnostic: pSTS (Model Using GPT Surprisal With the Effect of Token Size Regressed Out).
**Table S32:** Mixed‐effects model results: pSTS (Model Using GPT Surprisal With the Effect of Token Size Regressed Out).
**Table S33:** The effect of WO within each ADJ: pSTS (Model Using GPT Surprisal With the Effect of Token Size Regressed Out).
**Table S34:** Estimated slope of surprisal‐related fMRI signal increase in the pSTS (Model Using GPT Surprisal With the Effect of Token Size Regressed Out).
**Table S35:** Likelihood‐ratio tests for fixed‐effect structure: LIFS (Model Using GPT Surprisal With the Effect of Token Size Regressed Out).
**Table S36:** Fixed‐effect multicollinearity diagnostics: LIFS (Model Using GPT Surprisal With the Effect of Token Size Regressed Out).
**Table S37:** Convergence, singularity, and clustering diagnostic: LIFS (Model Using GPT Surprisal With the Effect of Token Size Regressed Out).
**Table S38:** Mixed‐effects model results: LIFS (Model Using GPT Surprisal With the Effect of Token Size Regressed Out).
**Table S39:** The effect of WO within each ADJ: LIFS (Model Using GPT Surprisal With the Effect of Token Size Regressed Out).
**Table S40:** Estimated slope of surprisal‐related fMRI signal increase in the LIFS (Model Using GPT Surprisal With the Effect of Token Size Regressed Out).

## Data Availability

The data that support the findings of this study are available on request from the corresponding author. The data are not publicly available due to privacy or ethical restrictions.

## Endnotes

^1^
This paper adopts Chomskyan generative linguistics for expository purposes. The discussions in this study may be made compatible with other frameworks such as Head‐Driven Phrase Structure Grammar (Pollard and Sag 1994), Tree‐Adjoining Grammar (Frank 2004), and Combinatory Categorial Grammar (Steedman 2001).

^2^
It is often assumed that the subject originates below TP and undergoes movement from its original position to its surface position. For the sake of simplicity, we do not consider this movement explicitly. Even if such subject movement is assumed, OSV sentences are still predicted to be representationally more complex than SOV sentences, because the object must undergo additional movement in the OSV sentences. See, for example, Koizumi and Tamaoka (2010, 668) for the relevant discussion.

^3^
Another possible derivation for the AS sentence with the VP adjunct (2a) is that the adjunct originally follows the object (i.e., SOAV) and undergoes scrambling over both the subject and the object (i.e., A_1_SOt_1_V). We do not consider this derivation here. This decision is motivated by processing considerations. It is widely assumed in the sentence‐processing literature that the human parser constructs simpler structures when the input and the grammar allow multiple derivations (e.g., Hawkins 2004; Marantz 2005; Pritchett and Whitman 1995). In the case of the ASOV sentence with the VP adjunct, taking SAOV as the base order yields a shorter filler–gap dependency than deriving ASOV from SOAV via scrambling of the adjunct over both the subject and the object. From a processing perspective, it is therefore natural to take SAOV as the underlying order for ASOV sentences with VP adjuncts.

^4^
Iwabuchi et al. (2019) labeled these ROIs as the pars opercularis (−52, 8, 28), the op9 (−46, 28, 13), and the pMTG (−54, −51, 6), according to which we set the search volumes for the ROI. However, we described the anatomical location of the ROIs differently. First, the searching of the individual maxima from the pars opercularis (−52, 8, 28) landed on LIFS. Similarly, the search from the op9 (−46, 28, 13) landed on LIFG, and the search from pMTG (−54, −51, 6) landed on pSTS.

^5^
We also found that a model including WORD ORDER and ADJUNCT (Signal Increase ~ WORD ORDER × ADJUNCT) fit the data significantly better than a model including GPT2‐surprisal alone (Signal Increase ~ GPT2‐surprisal), specifically in LIFG (logLik: −7932.7 vs. −7939.3, *X* ^2^ = 13.199, *p* = 0.010) and pSTS (logLik: −7894.2 vs. −7900.2, *X* ^2^ = 12.070, *p* = 0.017).
