# Supplementary material for: Unified Brain Mechanisms Underlying the Processing of Word Order Permutations of Arguments and Adjuncts in Sentences
Source: Hum Brain Mapp. 2026 Aug 13;47(11):e70604. doi: 10.1002/hbm.70604 (PMC13470681; doi:10.1002/hbm.70604)
Supplement: Supplementary file 1 [file HBM-47-e70604-s001.pdf]

Supplementary Data

1. Token size and negative log-probabilities (surprisal) calculated by GPT-2 Medium model

Values are reported as means (SDs) for each combination of ADJUNCT and WORD ORDER. t values were derived from post hoc pairwise comparisons of word order (AS vs SA) within each adjunct type based on a two-way repeated-measures ANOVA (within-item factors: WORD ORDER and ADJUNCT; Type III sums of squares; WORD ORDER × ADJUNCT interaction was significant). Simple-effect comparisons were computed with the emmeans function, and p values were adjusted across the three simple-effect comparisons using the Benjamini–Hochberg false discovery rate (FDR).

Table S1. Token size of each sentence.

	ASOV	SAOV	*t*	*p*
VP	9.85 (1.231)	9.85 (1.231)	-	-
TP	9.10 (0.955)	9.20 (0.966)	-2.082	.057
CP	9.20 (1.285)	9.35 (1.167)	-1.964	.057

Note: Because written Japanese does not use spaces to mark word boundaries (unlike English), the number of tokens produced by the tokenizer does not necessarily correspond to the number of words or phrases in a sentence. Dashes indicate that the comparison was not estimable.

Table S2. Sentence-level negative log-probabilities (surprisal) residualized for token size.

	ASOV	SAOV	*t*	*p*
VP	-1.044 (3.838)	-0.495 (3.892)	-2.234	.031
TP	-0.345 (3.945)	1.537 (4.476)	-4.050	< .001
CP	-1.917 (4.335)	2.263 (4.536)	-6.021	< .001

2. Results of behavioral data

Table S3. Within-Participant Mean Accuracy Across All Participants

		Mean	SD	Min	Max
VP	ASOV	0.948	0.124	0.333	1.00
	SAOV	0.957	0.113	0.500	1.00
TP	ASOV	0.950	0.077	0.750	1.00
	SAOV	0.920	0.163	0.143	1.00
CP	ASOV	0.915	0.165	0.250	1.00
	SAOV	0.927	0.126	0.500	1.00

Table S4. Within-Participant Mean Accuracy of Participants Included in the fMRI Analysis (Overall Accuracy ≥60%)

		Mean	SD	Min	Max
VP	ASOV	0.965	0.064	0.833	1.00
	SAOV	0.980	0.052	0.833	1.00
TP	ASOV	0.953	0.075	0.750	1.00
	SAOV	0.946	0.081	0.714	1.00
CP	ASOV	0.959	0.065	0.833	1.00
	SAOV	0.931	0.107	0.500	1.00

Table S5. Within-Participant Mean RT (Z-score) of Participants Included in the fMRI Analysis (Overall Accuracy ≥60%)

		Mean	SD	Min	Max
VP	ASOV	-0.014	0.243	-0.642	0.528
	SAOV	0.006	0.312	-0.724	0.546
TP	ASOV	0.089	0.438	-0.894	0.750
	SAOV	-0.043	0.438	-0.861	0.873
CP	ASOV	-0.039	0.325	-0.631	0.476
	SAOV	0.000	0.253	-0.507	0.532

3. Whole-brain analysis

3.1. The effect of experimental conditions

A whole-brain random-effects analysis revealed marginally significant clusters only for the CP & VP > TP contrast among the ADJUNCT effect contrasts. Greater activation was observed in one cluster encompassing the left supramarginal gyrus and left inferior parietal lobule (FDR-corrected $q = .057$), and in another cluster located in the left putamen (FDR-corrected $q = .066$). No other contrasts showed significant differences in activation.

Table S6. Brain regions identified by the contrast of CP & VP > TP.

Anatomical Region	$p_{\text{FWE-corr}}$	$q_{\text{FDR-corr}}$	k	Peak T	MNI coordinates		
Left supramarginal gyrus	.012	.057	128	4.95	-60	-52	26
Left inferior parietal lobule				4.30	-42	-49	35
				3.20	-48	-52	44
Left putamen	.027	.066	103	4.73	-15	8	5
				4.36	-27	-16	-7
				4.28	-21	-4	-1

Note: $p_{\text{FWE-corr}}$: Cluster-level FWE-corrected p. $q_{\text{FDR-corr}}$: Cluster-level FDR-corrected q. k: Cluster size.

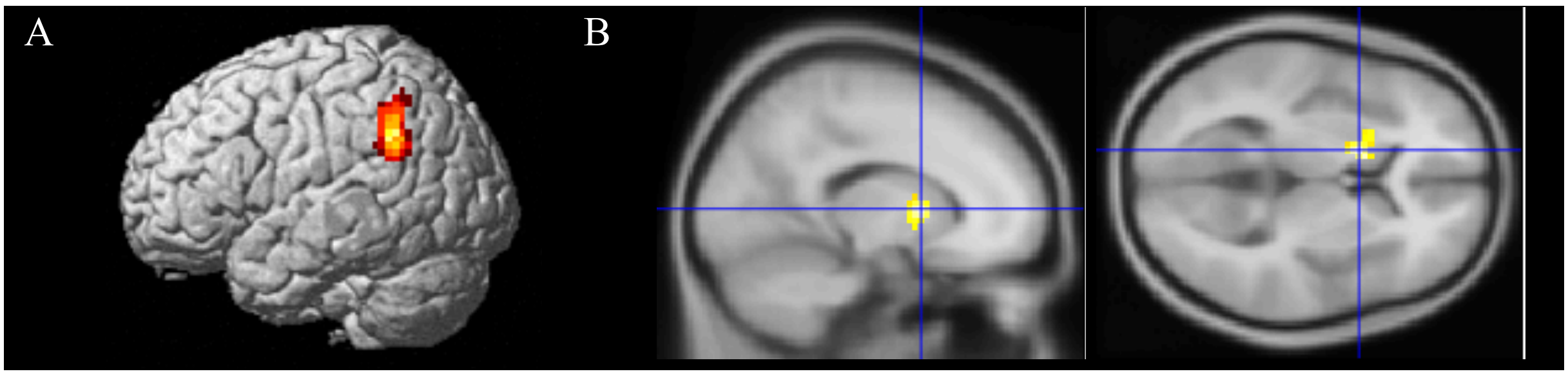

Figure S1. Results of a whole-brain random-effects analysis testing effects of ADJUNCT. Brain regions exhibiting marginally significant greater activation for CP and VP adjuncts than for TP adjuncts (CP & VP > TP, Cluster-level FWE-corrected *ps* < .05, Cluster-level FDR-corrected *qs* < .10) are overlaid on a rendered cortical surface (A) and on sagittal (B-left) and axial (B-right) slices (X = -15, Y = 8, Z = 5).

3.2. Brain activation during sentence comprehension relative to rest

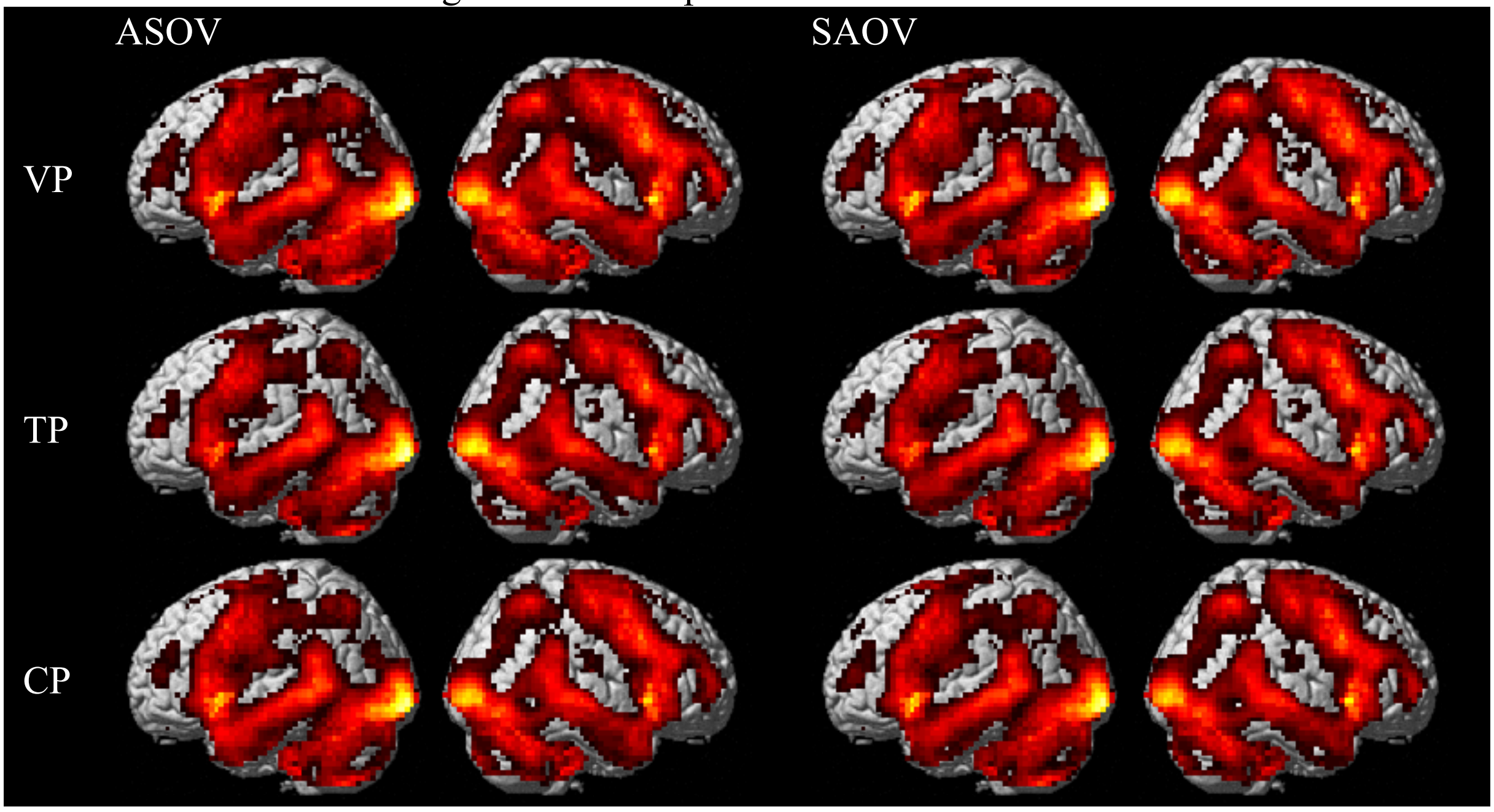

Figure S2. Brain images show results of whole-brain random effects analyses (Each condition > Rest, voxel-wise FDR-corrected *ps* < .05).

4. Trial time course (region of interest analysis)

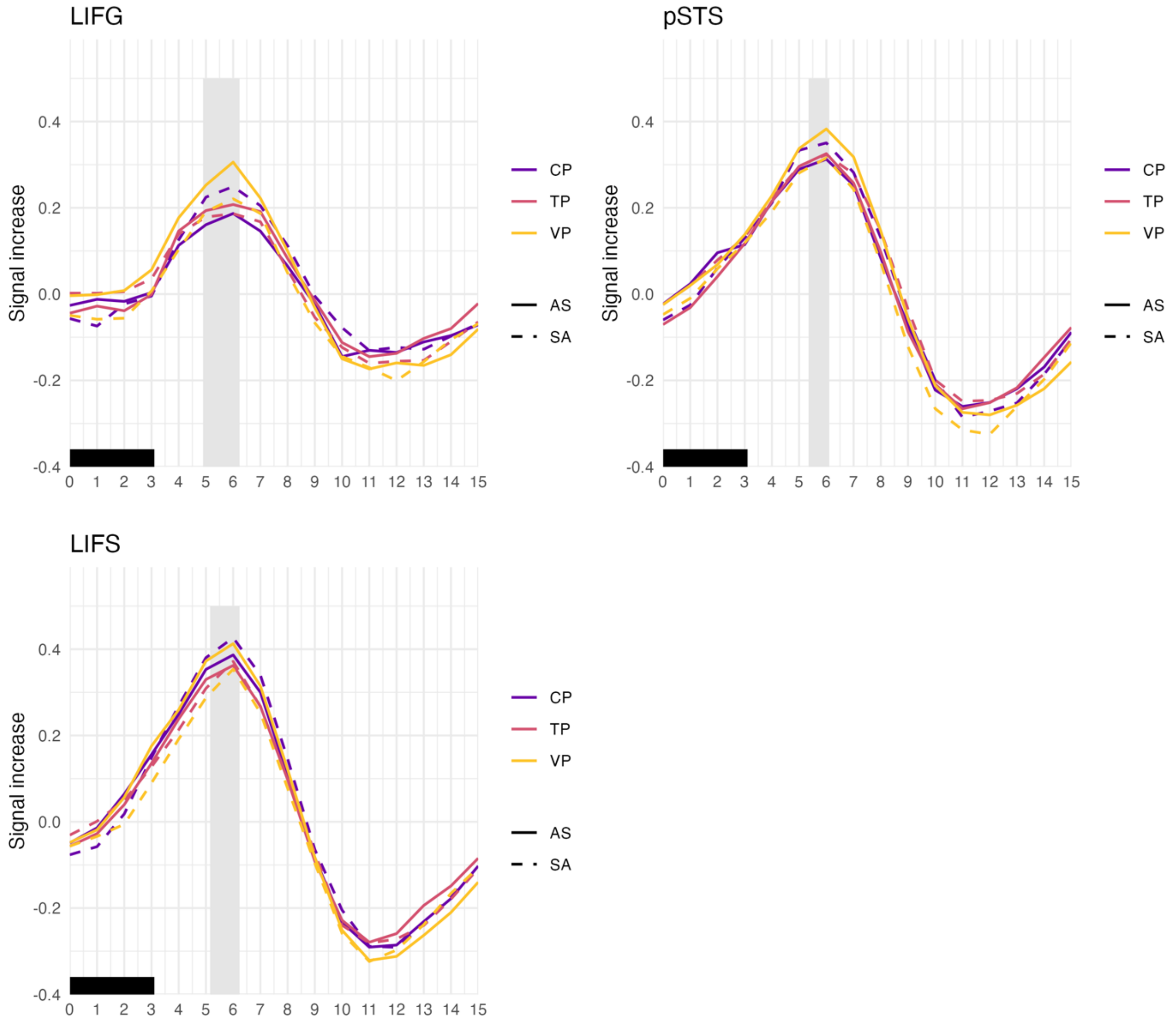

Figure S3. Mean trial time course (TTC) plots for each condition in each ROI. Time zero and the black rectangles at the bottom of the TTC plots correspond to the onset and duration of sentence presentation, respectively. The light gray bands indicate the 95% confidence intervals of mean peak latency within each ROI. LIFG: the left inferior frontal gyrus, LIFS: the left inferior frontal sulcus, pSTS: the posterior superior temporal sulcus. AS, Adjunct-Subject-Object-Verb word order; SA, Subject-Adjunct-Object-Verb word order.

5. Mixed-effects model results

We report the results in the main text for the models that define sentence-level surprisal as the sum of token-level surprisal values (Supplementary Data 5.1). However, the sum of token-level surprisal values within each sentence was positively correlated with sentence token size (Pearson's r = 0.286, 95% CI [0.166, 0.399], p < .0001). To ensure that the surprisal effect reflected contextual predictability rather than sentence length, we additionally conducted analyses using a length-residualized surprisal measure, obtained as the residuals from a linear model with summed surprisal as the dependent variable and token size as the predictor (Supplementary Data 5.2). Effect sizes for pairwise comparisons were expressed as standardized mean differences (Cohen's d), computed from the estimated marginal means using the residual standard deviation of the fitted mixed-effects model. For post hoc tests following significant interaction effects, reported *p* values were corrected for multiple comparisons using the false discovery rate (FDR) procedure.

5.1. Models defining sentence-level surprisal as the sum of token-level surprisal values

5.1.1. LIFG (surprisal: sum of token-level surprisal values)

The final model was:

*Signal Increase ~ WORD ORDER * ADJUNCT * GPT2-surprisal + (1 | participant) + (1 | item).*

Table S7. Likelihood-ratio tests for fixed-effect structure: LIFG

Fixed-effect structure	Log Likelihood	p (vs final model)
GPT2-surprisal	-7939.3	.003
*WORD ORDER * ADJUNCT + GPT2-surprisal*	-7932.6	.022
*WORD ORDER * ADJUNCT * GPT2-surprisal*	-7926.0	-

Table S8. Fixed-effect multicollinearity diagnostics of the final model: LIFG

Term	VIF	95% CI-LL	95% CI-UL
WORD ORDER	1.10	1.07	1.13
ADJUNCT	1.20	1.17	1.24
GPT2-surprisal	1.13	1.10	1.17
WORD ORDER : ADJUNCT	1.20	1.17	1.24
WORD ORDER : GPT2-surprisal	1.12	1.09	1.15
ADJUNCT : GPT2-surprisal	1.25	1.21	1.29
WORD ORDER : ADJUNCT : GPT2-surprisal	1.16	1.13	1.20

Note: CI-LL, Lower limit of the 95% confidence interval. CI-UL, Upper limit of the 95% confidence interval.

Table S9. Convergence, singularity, and clustering diagnostic of the final model: LIFG

Converged	Maximum absolute gradient	Singular	ICC (adjusted)	ICC (unadjusted)
TRUE	3.6843e-07	FALSE	0.097	0.097

ICC, Intraclass correlation coefficient

Table S10. Mixed-Effects Model Results: LIFG

Fixed Effect	β	95% CI-LL	95% CI-UL	t	p
(Intercept)	-0.1455	-0.2566	-0.0344	-2.5677	.0103
WO1	-0.0189	-0.0438	0.0060	-1.4874	.1370
ADJ1	-0.0028	-0.0390	0.0334	-0.1506	.8803
ADJ2	0.0159	-0.0186	0.0505	0.9047	.3656
GPT2-surp	0.0139	-0.0156	0.0434	0.9237	.3557
**WO1:ADJ1**	**-0.0618**	**-0.0984**	**-0.0253**	**-3.3168**	**.0009**
**WO1:ADJ2**	**0.0668**	**0.0323**	**0.1014**	**3.7946**	**.0001**
WO1:GPT2-surp	0.0021	-0.0233	0.0274	0.1588	.8739
**ADJ1:GPT2-surp**	**-0.0536**	**-0.0889**	**-0.0183**	**-2.9768**	**.0029**
**ADJ2:GPT2-surp**	**0.0558**	**0.0179**	**0.0938**	**2.8822**	**.0040**
WO1:ADJ1:GPT2-surp	-0.0094	-0.0448	0.0260	-0.5207	.6026
WO1:ADJ2:GPT2-surp	0.0251	-0.0122	0.0624	1.3199	.1869

Note: surp: sum of token-level surprisal values. WO, WORD ORDER. ADJ, ADJUNCT. WO1 (ASOV: 1, SAOV: -1), ADJ1 (CP: 1, TP: -1, VP: 0), ADJ2 (CP: 0, TP: -1, VP: 1). CI-LL, Lower limit of the 95% confidence interval. CI-UL, Upper limit of the 95% confidence interval.

Table S11. The effect of WO within each ADJ: LIFG

ADJ	Contrast	Cohen's d	95% CI-LL	95% CI-UL	z	p
**CP**	**AS - SA**	**-0.1743**	**-0.2736**	**-0.0750**	**-3.4425**	**.0018**
**VP**	**AS - SA**	**0.1036**	**0.0139**	**0.1932**	**2.2650**	**.0353**
TP	AS - SA	-0.0517	-0.1429	0.0396	-1.1101	.2670

Note: p values were adjusted using the FDR procedure; 95% confidence intervals were not adjusted for multiple comparisons. CI-LL, Lower limit of the 95% confidence interval. CI-UL, Upper limit of the 95% confidence interval.

The table of estimated slopes of surprisal-related fMRI signal increases in the left inferior frontal gyrus (LIFG) for each adjunct condition is presented in Table 4 of the main text.

5.1.2. pSTS (surprisal: sum of token-level surprisal values)

The final model was:

*Signal Increase ~ WORD ORDER * ADJUNCT * GPT2-surprisal + (1 | participant) + (1 | item).*

Table S12. Likelihood-ratio tests for fixed-effect structure: pSTS

Fixed-effect structure	Log Likelihood	p (vs final model)
GPT2-surprisal	-7900.2	.019
*WORD ORDER * ADJUNCT + GPT2-surprisal*	-7894.1	.102
*WORD ORDER * ADJUNCT * GPT2-surprisal*	-7889.5	-

Table S13. Fixed-effect multicollinearity diagnostics: pSTS

Term	VIF	95% CI-LL	95% CI-UL
WORD ORDER	1.10	1.07	1.13
ADJUNCT	1.20	1.17	1.24
GPT2-surprisal	1.13	1.10	1.17
WORD ORDER : ADJUNCT	1.20	1.17	1.24
WORD ORDER : GPT2-surprisal	1.12	1.09	1.15
ADJUNCT : GPT2-surprisal	1.25	1.21	1.29
WORD ORDER : ADJUNCT : GPT2-surprisal	1.16	1.13	1.20

Note: CI-LL, Lower limit of the 95% confidence interval. CI-UL, Upper limit of the 95% confidence interval.

Table S14. Convergence, singularity, and clustering diagnostic: pSTS

Converged	Maximum absolute gradient	Singular	ICC (adjusted)	ICC (unadjusted)
TRUE	4.145461e-07	FALSE	0.046	0.046

ICC, Intraclass correlation coefficient

Table S15. Mixed-Effects Model Results: pSTS

Fixed Effect	β	95% CI-LL	95% CI-UL	t	p
(Intercept)	0.0793	0.0028	0.1558	2.0325	.0422
WO1	-0.0079	-0.0326	0.0169	-0.6217	.5342
ADJ1	0.0363	0.0003	0.0723	1.9742	.0484
ADJ2	-0.0046	-0.0390	0.0298	-0.2637	.7920
GPT2-surp	-0.0012	-0.0286	0.0262	-0.0872	.9305
**WO1:ADJ1**	**-0.0540**	**-0.0902**	**-0.0178**	**-2.9228**	**.0035**
**WO1:ADJ2**	**0.0541**	**0.0198**	**0.0885**	**3.0860**	**.0020**
WO1:GPT2-surp	0.0104	-0.0148	0.0357	0.8125	.4166
**ADJ1:GPT2-surp**	**-0.0510**	**-0.0860**	**-0.0160**	**-2.8579**	**.0043**
ADJ2:GPT2-surp	0.0312	-0.0063	0.0687	1.6321	.1027
WO1:ADJ1:GPT2-surp	-0.0058	-0.0408	0.0293	-0.3223	.7472
WO1:ADJ2:GPT2-surp	0.0141	-0.0229	0.0512	0.7471	.4550

Note: surp: sum of token-level surprisal values. WO, WORD ORDER. ADJ, ADJUNCT. WO1 (ASOV: 1, SAOV: -1), ADJ1 (CP: 1, TP: -1, VP: 0), ADJ2 (CP: 0, TP: -1, VP: 1). CI-LL, Lower limit of the 95% confidence interval. CI-UL, Upper limit of the 95% confidence interval.

Table S16. The effect of WO within each ADJ: pSTS

ADJ	Contrast	Cohen's d	95% CI-LL	95% CI-UL	z	p
**CP**	**AS - SA**	**-0.1340**	**-0.2327**	**-0.0353**	**-2.6624**	**.0234**
**VP**	**AS - SA**	**0.1003**	**0.0107**	**0.1899**	**2.1949**	**.0423**
TP	AS - SA	-0.0174	-0.1085	0.0736	-0.3749	.7077

Note: p values were adjusted using the FDR procedure; 95% confidence intervals were not adjusted for multiple comparisons. CI-LL, Lower limit of the 95% confidence interval. CI-UL, Upper limit of the 95% confidence interval.

Table S17. Estimated slope of surprisal-related fMRI signal increase in the pSTS.

ADJ	Std. slope	95% CI-LL	95% CI-UL	z	p
CP	-0.0522	-0.0951	-0.0094	-2.3879	.0507
VP	0.0300	-0.0190	0.0789	1.2007	.3448
TP	0.0186	-0.0248	0.0620	0.8397	.4011

Note: Surprisal: sum of token-level surprisal values. p values were adjusted using the FDR procedure; 95% confidence intervals were not adjusted for multiple comparisons. CI-LL, Lower limit of the 95% confidence interval. CI-UL, Upper limit of the 95% confidence interval.

5.1.3. LIFS (surprisal: sum of token-level surprisal values)

The final model was:

*Signal Increase ~ WORD ORDER * ADJUNCT * GPT2-surprisal + (1 | participant).*

Table S18. Likelihood-ratio tests for fixed-effect structure: LIFS

Fixed-effect structure	Log Likelihood	p (vs final model)
GPT2-surprisal	-8693.3	.003
*WORD ORDER * ADJUNCT + GPT2-surprisal*	-8689.7	.002
*WORD ORDER * ADJUNCT * GPT2-surprisal*	-8680.1	-

Table S19. Fixed-effect multicollinearity diagnostics: LIFS

Term	VIF	95% CI-LL	95% CI-UL
WORD ORDER	1.10	1.07	1.13
ADJUNCT	1.20	1.17	1.24
GPT2-surprisal	1.13	1.10	1.17
WORD ORDER : ADJUNCT	1.20	1.17	1.24
WORD ORDER : GPT2-surprisal	1.12	1.09	1.15
ADJUNCT : GPT2-surprisal	1.25	1.21	1.29
WORD ORDER : ADJUNCT : GPT2-surprisal	1.16	1.13	1.20

Note: CI-LL, Lower limit of the 95% confidence interval. CI-UL, Upper limit of the 95% confidence interval.

Table S20. Convergence, singularity, and clustering diagnostic: LIFS

Converged	Maximum absolute gradient	Singular	ICC (adjusted)	ICC (unadjusted)
TRUE	1.845312e-08	FALSE	0.066	0.066

ICC, Intraclass correlation coefficient

Table S21. Mixed-Effects Model Results: LIFS

Fixed Effect	β	95% CI-LL	95% CI-UL	t	p
(Intercept)	-0.0007	-0.1050	0.1037	-0.0125	.9901
WO1	-0.0201	-0.0484	0.0083	-1.3887	.1650
ADJ1	-0.0088	-0.0499	0.0324	-0.4180	.6760
ADJ2	0.0234	-0.0160	0.0627	1.1653	.2440
GPT2-surp	-0.0030	-0.0317	0.0258	-0.2040	.8384
**WO1:ADJ1**	**-0.0641**	**-0.1053**	**-0.0230**	**-3.0543**	**.0023**
**WO1:ADJ2**	**0.0620**	**0.0226**	**0.1013**	**3.0884**	**.0020**
WO1:GPT2-surp	0.0017	-0.0270	0.0305	0.1176	.9064
**ADJ1:GPT2-surp**	**-0.0810**	**-0.1208**	**-0.0413**	**-3.9971**	**.0001**
**ADJ2:GPT2-surp**	**0.0456**	**0.0033**	**0.0879**	**2.1135**	**.0346**
WO1:ADJ1:GPT2-surp	-0.0235	-0.0632	0.0163	-1.1576	.2471
WO1:ADJ2:GPT2-surp	0.0324	-0.0067	0.0715	1.6224	.1048

Note: surp: sum of token-level surprisal values. WO, WORD ORDER. ADJ, ADJUNCT. WO1 (ASOV: 1, SAOV: -1), ADJ1 (CP: 1, TP: -1, VP: 0), ADJ2 (CP: 0, TP: -1, VP: 1). CI-LL, Lower limit of the 95% confidence interval. CI-UL, Upper limit of the 95% confidence interval.

Table S22. The effect of WO within each ADJ: LIFS

ADJ	Contrast	Cohen's d	95% CI-LL	95% CI-UL	z	p
**CP**	**AS - SA**	**-0.1595**	**-0.2576**	**-0.0614**	**-3.1889**	**.0042**
VP	AS - SA	0.0794	-0.0102	0.1690	1.7376	.1235
TP	AS - SA	-0.0339	-0.1247	0.0570	-0.7310	.4648

Note: p values were adjusted using the FDR procedure; 95% confidence intervals were not adjusted for multiple comparisons. CI-LL, Lower limit of the 95% confidence interval. CI-UL, Upper limit of the 95% confidence interval.

The table of estimated slopes of surprisal-related fMRI signal increases in the left inferior frontal sulcus (LIFS) for each adjunct condition is presented in Table 5 of the main text.

5.2. Models defining sentence-level surprisal as the residuals from regressing summed token-level surprisal on token size

5.2.1. LIFG (surprisal: residuals from regressing summed token-level surprisal on token size)

The final model was:

*Signal Increase ~ WORD ORDER * ADJUNCT * GPT2-surprisal + (1 | participant) + (1 | item).*

Table S23. Likelihood-ratio tests for fixed-effect structure: LIFG (Model Using GPT Surprisal With the Effect of Token Size Regressed Out).

Fixed-effect structure	Log Likelihood	p (vs final model)
GPT2-surprisal	-7938.8	.006
*WORD ORDER * ADJUNCT + GPT2-surprisal*	-7932.2	.045
*WORD ORDER * ADJUNCT * GPT2-surprisal*	-7926.6	-

Table S24. Fixed-effect multicollinearity diagnostics: LIFG (Model Using GPT Surprisal With the Effect of Token Size Regressed Out).

Term	VIF	95% CI-LL	95% CI-UL
WORD ORDER	1.12	1.09	1.16
ADJUNCT	1.26	1.22	1.30
GPT2-surprisal	1.20	1.17	1.24
WORD ORDER : ADJUNCT	1.28	1.24	1.32
WORD ORDER : GPT2-surprisal	1.13	1.11	1.17
ADJUNCT : GPT2-surprisal	1.27	1.24	1.32
WORD ORDER : ADJUNCT : GPT2-surprisal	1.21	1.17	1.25

Note: CI-LL, Lower limit of the 95% confidence interval. CI-UL, Upper limit of the 95% confidence interval.

Table S25. Convergence, singularity, and clustering diagnostic: LIFG (Model Using GPT Surprisal With the Effect of Token Size Regressed Out).

Converged	Maximum absolute gradient	Singular	ICC (adjusted)	ICC (unadjusted)
TRUE	1.771981e-07	FALSE	0.097	0.097

ICC, Intraclass correlation coefficient

Table S26. Mixed-Effects Model Results: LIFG (Model Using GPT Surprisal With the Effect of Token Size Regressed Out).

Fixed Effect	β	95% CI-LL	95% CI-UL	t	p
(Intercept)	-0.1430	-0.2541	-0.0320	-2.5253	.0116
WO1	-0.0153	-0.0404	0.0098	-1.1968	.2314
ADJ1	-0.0050	-0.0413	0.0313	-0.2713	.7862
ADJ2	0.0217	-0.0134	0.0568	1.2111	.2259
GPT2-surp (residualized)	0.0209	-0.0088	0.0507	1.3790	.1679
**WO1:ADJ1**	**-0.0590**	**-0.0956**	**-0.0223**	**-3.1543**	**.0016**
**WO1:ADJ2**	**0.0676**	**0.0326**	**0.1027**	**3.7829**	**.0002**
WO1:GPT2-surp (residualized)	0.0014	-0.0242	0.0269	0.1063	.9154
**ADJ1:GPT2-surp (residualized)**	**-0.0478**	**-0.0831**	**-0.0124**	**-2.6480**	**.0081**
**ADJ2:GPT2-surp (residualized)**	**0.0478**	**0.0098**	**0.0858**	**2.4650**	**.0137**
WO1:ADJ1:GPT2-surp (residualized)	-0.0105	-0.0458	0.0248	-0.5834	.5597
WO1:ADJ2:GPT2-surp (residualized)	0.0313	-0.0061	0.0686	1.6407	.1009

Note: surp: residuals from regressing summed token-level surprisal on token size. WO, WORD ORDER. ADJ, ADJUNCT. WO1 (ASOV: 1, SAOV: -1), ADJ1 (CP: 1, TP: -1, VP: 0), ADJ2 (CP: 0, TP: -1, VP: 1). CI-LL, Lower limit of the 95% confidence interval. CI-UL, Upper limit of the 95% confidence interval.

Table S27. The effect of WO within each ADJ: LIFG (Model Using GPT Surprisal With the Effect of Token Size Regressed Out).

ADJ	Contrast	Cohen's d	95% CI-LL	95% CI-UL	z	p
**CP**	**AS - SA**	**-0.1604**	**-0.2595**	**-0.0613**	**-3.1737**	**.0045**
**VP**	**AS - SA**	**0.1129**	**0.0214**	**0.2045**	**2.4176**	**.0234**
TP	AS - SA	-0.0518	-0.1437	0.0400	-1.1057	.2688

Note: p values were adjusted using the FDR procedure; 95% confidence intervals were not adjusted for multiple comparisons. CI-LL, Lower limit of the 95% confidence interval. CI-UL, Upper limit of the 95% confidence interval.

Table S28. Estimated slope of surprisal-related fMRI signal increase in the LIFG (Model Using GPT Surprisal With the Effect of Token Size Regressed Out).

ADJ	Std. slope	95% CI-LL	95% CI-UL	z	p
CP	-0.0268	-0.0706	0.0169	-1.2020	.3441
**VP**	**0.0687**	**0.0180**	**0.1194**	**2.6574**	**.0237**
TP	0.0209	-0.0259	0.0677	0.8760	.3810

Note: Surprisal: residuals from regressing summed token-level surprisal on token size. p values were adjusted using the FDR procedure; 95% confidence intervals were not adjusted for multiple comparisons. CI-LL, Lower limit of the 95% confidence interval. CI-UL, Upper limit of the 95% confidence interval.

5.2.2. pSTS (surprisal: residuals from regressing summed token-level surprisal on token size)

The final model was:

*Signal Increase ~ WORD ORDER * ADJUNCT * GPT2-surprisal + (1 | participant) + (1 | item).*

Table S29. Likelihood-ratio tests for fixed-effect structure: pSTS (Model Using GPT Surprisal With the Effect of Token Size Regressed Out).

Fixed-effect structure	Log Likelihood	p (vs final model)
GPT2-surprisal	-7900.2	.021
*WORD ORDER * ADJUNCT + GPT2-surprisal*	-7894.2	.111
*WORD ORDER * ADJUNCT * GPT2-surprisal*	-7889.7	-

Table S30. Fixed-effect multicollinearity diagnostics: pSTS (Model Using GPT Surprisal With the Effect of Token Size Regressed Out).

Term	VIF	95% CI-LL	95% CI-UL
WORD ORDER	1.12	1.09	1.16
ADJUNCT	1.25	1.22	1.29
GPT2-surprisal	1.18	1.15	1.21
WORD ORDER : ADJUNCT	1.26	1.23	1.30
WORD ORDER : GPT2-surprisal	1.13	1.11	1.17
ADJUNCT : GPT2-surprisal	1.27	1.23	1.31
WORD ORDER : ADJUNCT : GPT2-surprisal	1.20	1.17	1.24

Note: CI-LL, Lower limit of the 95% confidence interval. CI-UL, Upper limit of the 95% confidence interval.

Table S31. Convergence, singularity, and clustering diagnostic: pSTS (Model Using GPT Surprisal With the Effect of Token Size Regressed Out).

Converged	Maximum absolute gradient	Singular	ICC (adjusted)	ICC (unadjusted)
TRUE	1.357236e-07	FALSE	0.046	0.046

ICC, Intraclass correlation coefficient

Table S32. Mixed-Effects Model Results: pSTS (Model Using GPT Surprisal With the Effect of Token Size Regressed Out).

Fixed Effect	β	95% CI-LL	95% CI-UL	t	p
(Intercept)	0.0807	0.0042	0.1572	2.0677	.0387
WO1	-0.0066	-0.0316	0.0184	-0.5152	.6064
ADJ1	0.0368	0.0007	0.0730	1.9966	.0459
ADJ2	-0.0026	-0.0376	0.0323	-0.1468	.8833
GPT2-surp (residualized)	0.0018	-0.0259	0.0294	0.1267	.8992
**WO1:ADJ1**	**-0.0538**	**-0.0901**	**-0.0175**	**-2.9027**	**.0037**
**WO1:ADJ2**	**0.0558**	**0.0209**	**0.0907**	**3.1321**	**.0017**
WO1:GPT2-surp (residualized)	0.0087	-0.0167	0.0341	0.6735	.5007
**ADJ1:GPT2-surp (residualized)**	**-0.0503**	**-0.0853**	**-0.0153**	**-2.8182**	**.0048**
ADJ2:GPT2-surp (residualized)	0.0277	-0.0098	0.0653	1.4480	.1477
WO1:ADJ1:GPT2-surp (residualized)	-0.0042	-0.0392	0.0308	-0.2373	.8124
WO1:ADJ2:GPT2-surp (residualized)	0.0160	-0.0212	0.0531	0.8428	.3994

Note: surp: residuals from regressing summed token-level surprisal on token size. WO, WORD ORDER. ADJ, ADJUNCT. WO1 (ASOV: 1, SAOV: -1), ADJ1 (CP: 1, TP: -1, VP: 0), ADJ2 (CP: 0, TP: -1, VP: 1). CI-LL, Lower limit of the 95% confidence interval. CI-UL, Upper limit of the 95% confidence interval.

Table S33. The effect of WO within each ADJ: pSTS (Model Using GPT Surprisal With the Effect of Token Size Regressed Out).

ADJ	Contrast	Cohen's d	95% CI-LL	95% CI-UL	z	p
**CP**	**AS - SA**	**-0.1308**	**-0.2293**	**-0.0322**	**-2.6017**	**.0279**
**VP**	**AS - SA**	**0.1066**	**0.0151**	**0.1982**	**2.2837**	**.0336**
TP	AS - SA	-0.0186	-0.1102	0.0731	-0.3967	.6916

Note: p values were adjusted using the FDR procedure; 95% confidence intervals were not adjusted for multiple comparisons. CI-LL, Lower limit of the 95% confidence interval. CI-UL, Upper limit of the 95% confidence interval.

Table S34. Estimated slope of surprisal-related fMRI signal increase in the pSTS (Model Using GPT Surprisal With the Effect of Token Size Regressed Out).

ADJ	Std. slope	95% CI-LL	95% CI-UL	z	p
CP	-0.0485	-0.0909	-0.0061	-2.2429	.0747
VP	0.0295	-0.0193	0.0783	1.1848	.2898
TP	0.0244	-0.0208	0.0695	1.0586	.2898

Note: Surprisal: residuals from regressing summed token-level surprisal on token size. p values were adjusted using the FDR procedure; 95% confidence intervals were not adjusted for multiple comparisons. CI-LL, Lower limit of the 95% confidence interval. CI-UL, Upper limit of the 95% confidence interval.

5.2.3. LIFS (surprisal: residuals from regressing summed token-level surprisal on token size)
The final model was:
*Signal Increase ~ WORD ORDER * ADJUNCT * GPT2-surprisal + (1 | participant).*

Table S35. Likelihood-ratio tests for fixed-effect structure: LIFS (Model Using GPT Surprisal With the Effect of Token Size Regressed Out).

Fixed-effect structure	Log Likelihood	p (vs final model)
GPT2-surprisal	-8693.3	.006
*WORD ORDER * ADJUNCT + GPT2-surprisal*	-8689.7	.003
*WORD ORDER * ADJUNCT * GPT2-surprisal*	-8680.9	-

Table S36. Fixed-effect multicollinearity diagnostics: LIFS (Model Using GPT Surprisal With the Effect of Token Size Regressed Out).

Term	VIF	95% CI-LL	95% CI-UL
WORD ORDER	1.12	1.09	1.15
ADJUNCT	1.24	1.21	1.29
GPT2-surprisal	1.15	1.12	1.19
WORD ORDER : ADJUNCT	1.24	1.21	1.29
WORD ORDER : GPT2-surprisal	1.14	1.11	1.17
ADJUNCT : GPT2-surprisal	1.26	1.23	1.31
WORD ORDER : ADJUNCT : GPT2-surprisal	1.20	1.17	1.24

Note: CI-LL, Lower limit of the 95% confidence interval. CI-UL, Upper limit of the 95% confidence interval.

Table S37. Convergence, singularity, and clustering diagnostic: LIFS (Model Using GPT Surprisal With the Effect of Token Size Regressed Out).

Converged	Maximum absolute gradient	Singular	ICC (adjusted)	ICC (unadjusted)
TRUE	1.983106e-08	FALSE	0.066	0.066

ICC, Intraclass correlation coefficient

Table S38. Mixed-Effects Model Results: LIFS (Model Using GPT Surprisal With the Effect of Token Size Regressed Out).

Fixed Effect	β	95% CI-LL	95% CI-UL	t	p
(Intercept)	-0.0004	-0.1047	0.1039	-0.0079	.9937
WO1	-0.0173	-0.0459	0.0112	-1.1904	.2340
ADJ1	-0.0094	-0.0507	0.0319	-0.4473	.6547
ADJ2	0.0268	-0.0131	0.0667	1.3165	.1880
GPT2-surp (residualized)	-0.0021	-0.0310	0.0269	-0.1389	.8895
**WO1:ADJ1**	**-0.0631**	**-0.1044**	**-0.0218**	**-2.9964**	**.0027**
**WO1:ADJ2**	**0.0635**	**0.0235**	**0.1034**	**3.1166**	**.0018**
WO1:GPT2-surp (residualized)	-0.0011	-0.0301	0.0279	-0.0764	.9391
**ADJ1:GPT2-surp (residualized)**	**-0.0766**	**-0.1163**	**-0.0369**	**-3.7846**	**.0002**
ADJ2:GPT2-surp (residualized)	0.0371	-0.0053	0.0795	1.7156	.0863
WO1:ADJ1:GPT2-surp (residualized)	-0.0285	-0.0682	0.0112	-1.4082	.1591
WO1:ADJ2:GPT2-surp (residualized)	0.0370	-0.0022	0.0762	1.8481	.0646

Note: surp: residuals from regressing summed token-level surprisal on token size. WO, WORD ORDER. ADJ, ADJUNCT. WO1 (ASOV: 1, SAOV: -1), ADJ1 (CP: 1, TP: -1, VP: 0), ADJ2 (CP: 0, TP: -1, VP: 1). CI-LL, Lower limit of the 95% confidence interval. CI-UL, Upper limit of the 95% confidence interval.

Table S39. The effect of WO within each ADJ: LIFS (Model Using GPT Surprisal With the Effect of Token Size Regressed Out).

ADJ	Contrast	Cohen's d	95% CI-LL	95% CI-UL	z	p
**CP**	**AS - SA**	**-0.1524**	**-0.2503**	**-0.0545**	**-3.0520**	**.0069**
VP	AS - SA	0.0874	-0.0041	0.1788	1.8718	.0918
TP	AS - SA	-0.0335	-0.1249	0.0580	-0.7169	.4734

Note: p values were adjusted using the FDR procedure; 95% confidence intervals were not adjusted for multiple comparisons. CI-LL, Lower limit of the 95% confidence interval. CI-UL, Upper limit of the 95% confidence interval.

Table S40. Estimated slope of surprisal-related fMRI signal increase in the LIFS (Model Using GPT Surprisal With the Effect of Token Size Regressed Out).

ADJ	Std. slope	95% CI-LL	95% CI-UL	z	p
**CP**	**-0.0787**	**-0.1257**	**-0.0317**	**-3.2830**	**.0030**
VP	0.0351	-0.0186	0.0887	1.2816	.2000
TP	0.0375	-0.0123	0.0872	1.4761	.2000

Note: Surprisal: residuals from regressing summed token-level surprisal on token size. p values were adjusted using the FDR procedure; 95% confidence intervals were not adjusted for multiple comparisons. CI-LL, Lower limit of the 95% confidence interval. CI-UL, Upper limit of the 95% confidence interval.
